# Pathology and pathogenesis of bluetongue virus serotype 24 during experimental infection in native sheep

**DOI:** 10.3389/fcimb.2026.1710415

**Published:** 2026-03-10

**Authors:** S. Vineetha, M. Saminathan, Madhulina Maity, Gaurav K. Sharma, Mahajan Sonalika, Y. Krishnajyothi, Sanchay K. Biswas, A. Arun Prince Milton, M. S. L. Carvajal, Sushila Maan, Yashpal Singh Malik, K. P. Singh

**Affiliations:** 1 Centre for Animal Disease Research and Diagnosis (CADRAD), Indian Council of Agricultural Research (ICAR)-Indian Veterinary Research Institute, Bareilly, Uttar Pradesh, India; 2 Division of Virology, ICAR-Indian Veterinary Research Institute, Mukteshwar, Uttarakhand, India; 3 Division of Biological Standardization, Indian Council of Agricultural Research (ICAR)-Indian Veterinary Research Institute, Bareilly, Uttar Pradesh, India; 4 Department of Animal Husbandry, Veterinary Biological and Research Institute, Hyderabad, Telangana, India; 5 Division of Animal and Fisheries Sciences, ICAR-Research Complex for North Eastern Hill Region, Umiam, Meghalaya, India; 6 Department of Pathobiological Sciences, School of Veterinary Medicine, University of Wisconsin – Madison, Madison, WI, United States; 7 Department of Biotechnology, College of Veterinary Sciences, Lala Lajpat Rai University of Veterinary and Animal Sciences (LUVAS), Hisar, India

**Keywords:** bluetongue virus serotype 24, experimental infection, immune response, pathogenesis, pathology, sheep

## Abstract

**Introduction:**

Bluetongue virus (BTV) is a species of genus *Orbivirus* belonging to the *Sedoreoviridae* family. Bluetongue (BT) is endemic in India and responsible for causing significant economic losses to livestock farmers. In India, antibodies to BTV serotype 24 (BTV-24) have been reported in 2005; it was first isolated in 2010, and it caused several outbreaks in sheep during 2012–2014. The *in vivo* studies investigating the pathogenetic potential of various BTV serotypes in the susceptible host sheep are scarce. Furthermore, detailed investigations to elucidate the pathogenetic mechanisms of BTV-24 under experimental conditions in sheep are not available. Because of its impact on the livestock economy, the present study was undertaken for the first time to explore the infection kinetics, pathology, pathogenesis, and immune responses against the Indian isolate of BTV-24 in sheep under experimental conditions.

**Methods:**

Six native sheep were infected intradermally with BTV-24 at 10^6^ TCID_50_/mL concentration, and six sheep were inoculated with uninfected cell culture fluid. Animals were euthanized at 4, 7, 11, 16, 45, and 60 days post-inoculation (DPI). The sequential pathology, BTV localization by immunohistochemistry, BTV quantification by quantitative PCR (qPCR), immune cell kinetics [CD4^+^ and CD8^+^ T lymphocytes in peripheral blood mononuclear cells (PBMCs), prescapular lymph node (PSLN), and spleen] by fluorescence-activated cell sorting (FACS), and cytokine estimation by qRT-PCR were studied.

**Results:**

The BTV-24-infected animals showed pyrexia, conjunctival and oral mucosal congestion, cyanosis of tongue, serous to catarrhal nasal discharge, and viremia. Gross pathological lesions were observed in the lymph nodes, lungs, and kidneys, with the lymph nodes being enlarged, edematous, and hemorrhagic. Subintimal hemorrhage at the base of the pulmonary artery (pathognomonic lesion of BT) was observed at 7 DPI. Histopathological lesions were prominent in lymph nodes, spleen, heart, lungs, and cerebral endothelium. Severe hemosiderosis in spleen, and hemorrhages and hyalinization of tunica media in pulmonary artery at 7 DPI were observed. Development of clinical signs and gross and histopathological lesions in BTV-24-infected animals emphasized the moderate progression of disease and enhanced virulence of the serotype. Humoral immune response was significantly high at 5, 11, 16, 21, 45, and 60 DPI. Cell-mediated immune response-like kinetics of CD4^+^ and CD8^+^ T lymphocytes showed a sharp decline during the early stage and an increase of CD8^+^ T lymphocytes during later stages of infection. BTV antigen was detected consistently in tongue, thymus, trapezius muscle, heart, and pulmonary artery by immunohistochemistry and qPCR. Significant changes in the levels of cytokines [interferon-alpha (IFN-α), IFN-β, IFN-γ, interleukin-2 (IL-2), IL-12, and tumor necrosis factor-alpha (TNF-α)] and upregulated expression of apoptotic markers, B-cell lymphoma-2 (Bcl-2), and caspase-3 in the spleen and lymph nodes were correlated with peak viremia.

**Conclusion:**

The results of this study can be used to formulate effective preventive and control measures and to develop a suitable vaccine against BTV-24 to minimize economic losses.

## 1 Introduction

Bluetongue (BT) is an infectious, non-contagious vector-borne viral pathosis of ruminants caused by bluetongue virus (BTV), a prototype member of the genus *Orbivirus* in the family *Sedoreoviridae* (formerly *Reoviridae*). The disease is transmitted by biting midges of the genus *Culicoides*. The disease draws its name from the most recognizable of symptoms (BT) that an infected animal presents. The disease is also termed “malarial catarrhal fever” or “epizootic malignant catarrhal fever of sheep” (Saminathan et al., 2020). Until recently, 28 BTV serotypes have been described globally with the addition of some “atypical” serotypes, becoming a total of 36 BTV serotypes based on the differences in the genome segment-2 (Seg-2) sequence and its translated protein VP2 (Sun et al., 2016; Bumbarov et al., 2020; Caixeta et al., 2024). Because of the recent spread of BT into previously unaffected regions of the world and the incursion of few additional serotypes, BT has again risen in prominence as one of the important diseases of the 21st century (Saminathan et al., 2020). The clinical manifestations of the BT vary from asymptomatic to lethal outcome based on virus factors like serotype of BTV, passage history of the virus, and dose and route of inoculation; host factors such as age, species, breed, individual susceptibility and immune status, stress, and nutritional status of the host; and environmental factors such as solar irradiation, high temperature, and vector population (Schwartz-Cornil et al., 2008; Maclachlan et al., 2009; Saminathan et al., 2018). Virulence characteristics vary among field strains of BTV, even those of the same serotype due to the considerable genetic variability results from mutations in the different genome segments, and such changes could influence the biological properties of the virus, which might result in the variable expression of disease (Saminathan et al., 2020). Despite these facts, *in vivo* comparative studies focused on determining the differences in the pathogenetic potential of BTV serotypes in the susceptible host are scarce with few reports in sheep (Hamblin et al., 1998; Sanchez-Cordon et al., 2013).

Currently, BT is endemic in India. Among 28 serotypes of BTV, 23 serotypes (except 22 and 25–28) have been reported from India based on the presence of neutralizing antibodies and virus isolation (Singh et al., 2021). The virus thrives throughout tropical, subtropical, and temperate regions of the world, wherever competent vector populations predisposed to its dissemination exist. Bluetongue virus serotype 24 (BTV-24) was first reported from Africa (Nevill et al., 1992) and later isolated from America and the Mediterranean in the last decade (Brenner et al., 2010). Antibodies to BTV-24 have been reported from buffaloes in India (Chauhan et al., 2005; Saminathan et al., 2020), and the serotype was first isolated from India in 2010. BTV-24 was involved in several outbreaks in various states of India indicating the emergence and virulence of this serotype (Singh et al., 2021). Sequence analysis for Seg-2 of BTV-24 (IND2010/01) indicated that the virus is closely related to Western viruses; hence, the entry of BTV-24 to India was exotic to Australasia, which was a cause of concern (Krishnajyothi et al., 2016). The seroprevalence of BTV-24 in India was reported as 1.61% in Telangana and Andhra Pradesh during 2017–2018, which increased to 16.66% in Telangana during 2018–2019 (Reddy et al., 2010, Naresh et al., 2020; Putty et al., 2020).

The pentavalent inactivated adjuvanted vaccine containing BTV-1, -2, -10, -16, and -23 has been used for the control of BT in India (Naresh et al., 2020). Recent studies revealed that unpredictably; serotypes that are not there in the currently used pentavalent vaccine, i.e., BTV-24, -4, -5, -9, -12, and -21, have emerged with high prevalence rates in India (Naresh et al., 2020: Putty et al., 2020). Recent studies from our lab revealed that the currently used pentavalent vaccine in India did not give protection against BTV-24 and BTV-4 (data not published), which created important discussions about the revision of BTV serotypes in the current vaccine. Hence, there is a need to update or revise BTV serotypes present in the current BTV pentavalent vaccine with BTV-24 after knowing its virulence. The pathology and pathogenesis of Indian BTV-24 in the natural host sheep have not been studied yet and, thus, need to be determined for its inclusion in the existing pentavalent vaccine for control measures. Considering these critical gaps, the present study was conducted for the first time to investigate the infection kinetics, pathology, pathogenesis, and immune responses of BTV-24 infection in the natural host sheep.

## 2 Materials and methods

### 2.1 Bluetongue virus serotype 24 isolate

The BTV-24 isolate used in this study was isolated from sheep, which showed symptoms of fever and hyperemia of gums and tongue with a morbidity rate of 15%–20% and a case–fatality rate of 6.91% during the BT outbreak in 2010 in Medak district, Telangana State, India (Krishnajyothi et al., 2016). The virus isolate was kindly provided by the Veterinary Biological and Research Institute, Hyderabad, Telangana, India.

### 2.2 Propagation of BTV-24 isolate

The BTV-24 isolate (passage 7) was propagated in the insect *Culicoides sonorensis* (KC) cell line maintained in Schneider’s insect medium (Sigma-Aldrich, St. Louis, MO, USA) with 10% fetal calf serum (FCS) and antibiotic–antimycotic solution followed by cultivation in baby hamster kidney-21 (BHK-21) cells (clone-13, National Centre for Cell Science, Pune, Maharastra, India) at passage 30 maintained in modified Eagle’s medium (HiMedia Laboratories, Thane, Maharashtra, India) supplemented with 10% FCS and antibiotic–antimycotic solution containing 10,000 U of penicillin, 10 mg of streptomycin, and 25 µg of amphotericin B per milliliter (HiMedia Laboratories, Thane, Maharashtra, India) and subsequently in BHK-21 cells. The crude culture supernatant was collected and combined with the supernatant obtained after sonication of infected cells. This pooled supernatant was subjected to endpoint titration in BHK-21 cells. For long-term storage, the virus stock was maintained at −80 °C, and for short-term storage, it was maintained at 4 °C. All procedures were performed under BSL-2 containment.

### 2.3 Confirmation of BTV-24

The BTV-24 serotype was confirmed by PCR using self-designed serotype-specific forward: 5′-AGTGACCCACAATGGAGGAG-3′ and reverse: 5′-TGAGTGCGTCTACTATGCTACTT-3′ primers targeting the 195-bp product of VP2 gene (segment 2—gene accession number KX164150). The total RNA was extracted from the cell culture using TRIzol^®^ reagent as per the manufacturer’s recommendations. The purity of the RNA was analyzed in a NanoDrop^®^ ND-1000 spectrophotometer (Thermo Fisher Scientific, Wilmington, DE, USA). The reaction was carried out in Stratagene Mx3005P™ Multiplex QPCR using a One-Step RT-PCR kit (QIAGEN GmbH, Hilden, North Rhine-Westphalia, Germany). The PCR reaction mix (25 μL containing 10 pmol of forward and reverse primer) was prepared and subjected to a cycling condition of reverse transcription at 50°C for 30 min, initial PCR activation at 95°C for 15 min, template denaturation at 94°C, primer annealing at 62°C, extension at 72°C for 30 s, and final extension at 72°C for 10 min for 35 cycles. The amplicons were visualized on 1.5% agarose gel (Sigma-Aldrich, USA) prepared in 1 × TBE buffer containing ethidium bromide (0.5 μg/mL). The gel was visualized under a gel documentation system (Azure Biosystems-c300, USA) for 195-bp amplicons. The amplicon was purified using commercially available kits (QIAquick Gel Extraction Kit, Hilden, North Rhine-Westphalia, Germany). The gel-purified products were outsourced for sequencing from Eurofins Genomics India Ltd., Whitefield, Bangalore, using forward and reverse primers in a capillary sequencer.

### 2.4 Experimental animals

Twelve indigenous nondescript adult sheep of either sex aged between 2 and 3 years of approximately 30–40 kg were procured from livestock farmers after screening and negative for BTV antibodies using a competitive-enzyme linked immunosorbent assay (c-ELISA) kit (Bluetongue virus antibody cELISA test kit, VMRD Inc., Pullman, WA, USA). The sheep were housed in the insect-proof animal shed of the Centre for Animal Disease Research and Diagnosis (CADRAD), ICAR-Indian Veterinary Research Institute (ICAR-IVRI), Bareilly, Uttar Pradesh, India. The animals were provided feed, fodder, and water *ad libitum* throughout the experiment. This study was approved by IAEC (ICAR-IVRI), and all animal procedures were conducted in accordance with the Committee for the Purpose of Control and Supervision on Experiments on Animals (CPCSEA) guidelines, 2003 (Ethical Clearance Certificate’s Number 108/HRECC.FODM/VII/2017).

The animals were randomly assigned to groups. The animals of either sex were allotted to different groups irrespective of the sex. The animals were housed in the animal isolation facility of ICAR-IVRI for 28 days followed by acclimatization for 1 week at the Experimental Animal House Facility of the institute. The animals were screened for hemoparasites and antibodies against BTV prior to the study.

### 2.5 Experimental design

The BTV-infected and uninfected control animals were housed indoors separately. The uninfected control group with six sheep were administered 6 mL of mock infected cell culture fluid via the intradermal route at multiple sites. In the BTV-24-infected group, six sheep were inoculated with 6 mL of 1 × 10^6^ TCID_50_/mL of BTV-24 via the intradermal route at multiple sites in the right pre-scapular or neck region. The infection design, including viral dose and trial structure, was based on previously published experimental BTV infection studies that have demonstrated reliable establishment of viremia and clinical response under similar conditions (Umeshappa et al., 2010, 2011; Channappanavar et al., 2012; Singh et al., 2024). The selected inoculum dose has been widely used in earlier work to ensure consistent infection dynamics, and the replication number aligns with established experimental models for BTV pathogenesis (Singh et al., 2024). The mock-control group consisted of virus-free culture medium processed identically to the infected inoculum, thereby ensuring appropriate procedural and systemic comparability.

### 2.6 Monitoring of clinical signs

The rectal temperature and clinical signs of each animal were recorded throughout the experimental period of 45 days daily and up to 60 days weekly before 11 a.m. to minimize any diurnal fluctuations in the estimated parameters and scored using the clinical reaction index as described by Moulin et al. (2012) and Caporale et al. (2014) with slight modifications (**Supplementary Table 1**).

### 2.7 Sample collection

Blood samples were collected at 0, 1, 3, 5, 7, 9, 11, 14, 17, 21, 28, 45, and 60 days post-inoculation (DPI) to assess the humoral immune response and viremia. Approximately 4 mL of blood was collected at each time interval for various parameters. Viremia evaluation was detailed by including blood and peripheral blood mononuclear cells (PBMCs) in the sections mentioned. One sheep from each uninfected and BTV-24-infected group was pre-euthanized by administration of a combination of xylazine (0.22 mg/kg b. wt) and ketamine (11 mg/kg b. wt) intramuscularly followed by euthanasia by administering single intravenous injection at a dose of 40 mg/kg b. wt of thiopental sodium (Thiosol sodium 1 g, Neon Laboratories Ltd., Mumbai, Maharashtra, India) via the jugular vein at 4, 7, 11, 16, 45, and 60 DPI. The required volume of thiopental was injected reasonably rapidly. The death of the animal was ensured by checking the absence of eye reflexes and heartbeat and the appearance of glazed eyes following injection, and systematic postmortem examination was carried out.

The blood samples collected before euthanasia were used for the estimation of immune cells’ (CD4^+^ and CD8^+^ T lymphocytes) kinetics. The gross pathological lesions were graded (Umeshappa et al., 2010; Sanchez-Cordon et al., 2013; **Supplementary Table 2**). Representative triplicate samples from lungs, heart, pulmonary artery, prescapular lymph nodes (PSLNs), spleen, trapezius muscle, skin, tonsil, and tongue were collected in 10% neutral buffered formalin for histopathology and RNAlater^®^ (Ambion^®^, Austin, TX, USA) for RNA extraction. Spleen and PSLN were collected aseptically in RPMI-1640 medium (Sigma-Aldrich, St. Louis, MO, USA) in ice for the analysis of immune cells’ (CD4^+^ and CD8^+^ T lymphocytes) kinetics from the euthanized animals at 4, 7, 11, 16, 45, and 60 DPI.

### 2.8 Histopathology

The formalin-fixed tissues were cut into pieces of 2 to 3 mm thickness, washed with water, dehydrated in ascending grades of alcohol, and cleared in xylene. The cleared tissues were embedded in paraffin, and sections of 4–5 μm thickness were cut and stained with hematoxylin and eosin (H&E) as per the standard procedure (Luna, 1968). The histopathological lesion score was calculated semi-quantitatively by following the scoring system of Umeshappa et al. (2011) with slight modification (**Supplementary Table 3**).

### 2.9 Immunohistochemistry

The formalin-fixed tissue sections were taken on (3-aminopropyl)triethoxysilane (APES, Sigma-Aldrich, St. Louis, MO, USA)-coated slides. The sections were deparaffinized and rehydrated in graded alcohol followed by gently rinsing in distilled water. The antigen retrieval was performed by microwave in 10 mM tri-sodium citrate buffer (pH 6.0) for 15 min (three cycles of 5 min each) to unmask the antigenic sites. Then, the slides were washed three times with phosphate buffered saline (PBS, pH 7.4) for 5 min each. Endogenous peroxidase activity was blocked by incubating with freshly prepared 3% H_2_O_2_ in methanol for 30 min at room temperature (RT) in a dark chamber and washed in PBS (three times for 5 min each). Furthermore, the sections were incubated with 5% bovine serum albumin (BSA) in PBS for 1 h at RT in a humidified chamber for blocking of non-specific antigen binding sites. The primary antibody against BTV core antigen raised in rabbit at the Mukteshwar campus of the Indian Veterinary Research Institute (IVRI) was incubated on the sections at 1:20 dilution in 1% BSA in PBS overnight in a humidified chamber at 4 °C. Slides were incubated with biotinylated goat anti-rabbit IgG peroxidase conjugate (Sigma-Aldrich, St. Louis, MO, USA) at 1:200 dilution in 1% BSA (pH 7.4) for 1 h at 37 °C, followed by washing thrice in PBS for 5 min each. The slides were incubated with ImmPACT™ DAB peroxidase substrate (Vector Laboratories Inc., Burlingame, CA, USA) for 30–60 s to demonstrate immunolabeling. The slides were counterstained with Mayer’s hematoxylin. The sections were mounted with CC/Mount™ aqueous mounting medium (Sigma-Aldrich, St. Louis, MO, USA) and dried at RT. Sections prepared from known BTV-positive samples were used as positive control. In negative tissue control, tissues sections were incubated only with BSA instead of BTV primary antibody. The sections were examined under the microscope for positive signals. In the negative antibody control, tissue sections were treated identical to other slides and PBS was added instead of BTV antibody. Similarly, expression of caspase-3 was studied using rabbit anti-caspase-3 polyclonal (Santa Cruz Biotechnology, Inc. Dallas, TX, USA) as the primary antibody at 1:10 dilution and biotinylated goat anti-rabbit IgG peroxidase conjugate (Sigma-Aldrich, St. Louis, MO, USA) at 1:200 dilution as the secondary antibody.

### 2.10 Scoring the intensity of immunohistochemistry signals

To quantify the BTV-specific antigen semi-quantitatively, tissue sections were evaluated based on the number of positive cells for BTV antigen found in 10 high-power (40×) fields on a − to +++ scale as follows: −: absence of immunostaining; +: less than 10% cells positive for BTV antigen (weak); ++: 10%–50% cells positive (moderate); and +++: more than 50% cells positive (strong).

### 2.11 Estimation of humoral immunity

The development of group-specific antibodies against BTV was measured in the samples collected at different time intervals using the c-ELISA kit developed at Mukteswar Campus, ICAR-IVRI, Uttarakhand, India (Chand et al., 2017). The optical density (OD) was measured at a wavelength of 492 nm (Bio-Rad, Hercules, CA, USA). The percentage inhibition (PI) value for each sample was calculated from the OD of the test samples and the negative serum controls using the following formula. The test samples were considered positive, when the PI value of the sample is equal to or more than 50%.


$$\;\text{Percentage inhibition }\left(\text{PI}\right)=[100-[\frac{\text{Mean OD}492\text{ of test sample}}{\text{Mean OD}492\text{ of negative serum control}}]\times 100]$$

### 2.12 Estimation of cell-mediated immunity

#### 2.12.1 Isolation of peripheral blood mononuclear cells

The pooled blood samples were used for the isolation of PBMCs using Histopaque by density gradient centrifugation as described previously by Saminathan et al. (2020). Briefly, approximately 2 mL of blood was slowly layered over an equal volume of histopaque [Histopaque^®^ (Sigma-Aldrich, St. Louis, MO, USA) with a density of 1.077 g/mL] in a 15-mL centrifuge tube and centrifuged at 210*g* for 40 min, resulting in the separation of PBMCs at the plasma–histopaque interface. The PBMCs were separated carefully and washed twice with isotonic PBS (0.01 M, pH 7.3) at 375*g* for 10 min each. To remove the traces of red blood cells (RBCs), 1 mL of 1× RBC lysis buffer (HiMedia Laboratories, Pennsylvania, USA) was added and incubated for 10 min in ice and centrifuged at 2,000 rpm for 5 min. The PBMCs were used for the analysis of CD4^+^ and CD8^+^ T lymphocytes using fluorescence-activated cell sorting (FACS) and RNA extraction for cytokine genes expression studies.

#### 2.12.2 Isolation of lymphocytes and splenocytes

Splenocytes were separated from spleen by mincing into small pieces as described previously (Madhu et al., 2016). Aseptically collected spleen and PSLN from BTV-infected and uninfected control animals were homogenized and passed through a cell strainer (70 µm) to make a single-cell suspension. Single-cell suspension was washed twice with PBS (0.01 M, pH 7.3) and centrifuged at 580*g* for 5 min at 4°C. The cell pellet was suspended in 1 mL of RBC lysis buffer, incubated at 4°C for 10 min, and centrifuged at 580*g* for 5 min. Separated cells were washed twice in isotonic PBS and centrifuged at 375*g* for 5 min each. The isolated lymphocytes and splenocytes were used for the analysis of CD4^+^ and CD8^+^ T lymphocytes using FACS and RNA extraction for cytokine gene expression studies.

#### 2.12.3 Estimation of CD4^+^ and CD8^+^ T lymphocytes by FACS

The PBMCs from blood, lymphocytes from PSLN, and splenocytes from spleen were resuspended in 200 μL of stain buffer (FBS, BD Pharmingen™, New Jersey, USA). Cells were counted by the dye exclusion method in a Neubauer chamber after dilution at 1:10 dilution with trypan blue solution (0.01%) to adjust the cell concentration of at least 1 × 10^6^ cells per sample. Each 10 µl of mouse anti-sheep CD4:RPE-labeled (Bio-Rad Laboratories Inc., Watford, Hertfordshire WD17 1ET, United Kingdom) and mouse anti-sheep CD8:FITC-labeled antibodies (Bio-Rad Laboratories Inc., Watford, Hertfordshire WD17 1ET, United Kingdom) was added as per the manufacturer’s instruction. Then, samples were mixed and incubated at RT in the dark for 45 min. Data were acquired using flow cytometry (BD^®^ LSR II Flow Cytometer, BD Biosciences, Franklin Lakes, NJ, USA) and interpretation of data (10,000 events/sample) was done by comparing the data with the uninfected control animals using BD CellQuest™ Pro Software (BD Biosciences, San Jose, CA, USA).

#### 2.12.4 RNA extraction

Total RNA was extracted from the blood and tissue samples (skin, tongue, thymus, tonsil, trapezius muscle, spleen, PSLN, lungs, heart, and pulmonary artery) using TRIzol^®^ reagent (Invitrogen™, Thermo Fisher Scientific, Carlsbad, CA, USA) as per the manufacturer’s recommendations. The total RNA was treated with RNase-free DNase (Promega, Madison, Wisconsin, USA) followed by enzyme inactivation at 65 °C for 10 min to remove the possible traces of genomic DNA (Madhu et al., 2016). Purity of the RNA was analyzed in a NanoDrop^®^ ND-1000 spectrophotometer (Thermo Fisher Scientific, Wilmington, DE, USA) and the integrity of the RNA was tested by electrophoresis. The RNA pellet was stored at −80 °C until further use.

#### 2.12.5 Complementary DNA synthesis

Complementary DNA (cDNA) was synthesized from total RNA using the Verso cDNA Synthesis Kit (Thermo Fisher Scientific, Carlsbad, CA, USA) with random primers following the manufacturer’s instruction in the Thermocycler (Mastercycler Personal, Eppendorf, Hamburg, Germany). The synthesized cDNA was checked using β-actin-specific primers by polymerase chain reaction (PCR) using GoTaq^®^ Green Master Mix (Promega, Madison, Wisconsin, USA). The synthesized cDNA was stored at −20°C until used.

#### 2.12.6 Quantification of cytokine genes’ expression by quantitative RT-PCR

The expression of mRNA of different cytokines [interferon-alpha (IFN-α), IFN-β, IFN-γ, interleukin-2 (IL-2), IL-12, and tumor necrosis factor-alpha (TNF-α)] and apoptotic markers [B-cell lymphoma-2 (Bcl-2) and caspase-3] was quantified in the PBMCs, PSLN, and spleen of the BTV-infected and uninfected control animals at specified time intervals by comparing with GAPDH as the internal reference gene (Umeshappa et al., 2010) using QuantiFast^®^ SYBR^®^ Green PCR master mix (Qiagen, Maryland, USA) in a Stratagene Mx3000P™ Multiplex Quantitative PCR (QPCR) system (Agilent Technologies, Santa Clara, CA, USA). The details of the primers used for quantification of different cytokine genes in sheep are mentioned in **Table 1**. Reactions were performed in triplicate for each gene of interest; a dissociation curve was generated after PCR amplification and analyzed to determine the specificity of the PCR reaction. The comparative CT (2^−ΔΔ^*^C^*^t^) method was used to calculate the changes in the gene expression as a relative fold change between different groups (Livak and Schmittgen, 2001).

**Table 1:** Details of oligonucleotide primers used for quantification of different cytokine genes in sheep.

Gene	Primer sequence	Primer length (bp)	Product length (bp)	Annealing temperature (°C)	Reference
GAPDH	F: 5′-ATCTCGCTCCTGGAAGATG-3′	19	227	60	Puech et al. (2015)
	R: 5′-TCGGAGTGAACGGATTCG-3′	18			
IFN-α	F: 5′-CAGACCATCTCTGTGCTCC-3′	19	216	55	Umeshappa et al. (2010)
	R: 5′-GTGTTTCCTCACAGCCAGG-3′	19			
IFN-β	F: 5′-CCAGATGGTTCTCCTGCTGTGT-3′	22	216	60	bin Tarif et al. (2012)
	R: 5′-GACCAATACGGCATCTTCCTTC-3′	22			
IFN-γ	F: 5′-GATAACCAGGTCATTCAAAGG-3′	21	222	60	Umeshappa et al. (2010)
	R: 5′- GAGATTCTGACTTCTCTTCC-3′	20			
IL-2	F: 5′-GCTCCAAGCAAAAACCTGAA-3′	20	110	60	Wani et al. (2018)
	R: 5′-CAGCCTTTACTGTCGCATCA-3′	20			
IL-12	F: 5′-CGTGATGGAAGCTGTGCAC-3′	19	211	60	Umeshappa et al. (2010)
	R: 5′-CTTTCCCTGGACCTGAACAC-3′	20			
TNF-α	F: 5′-CTTCAACAGGCCTCTGGTTC-3′	20	111	60	Puech et al. (2015)
	R: 5′-GGACCTGCGAGTAGATGAGG-3′	20			
Bcl-2	F: 5′-TTCGCCGAGATGTCCAGT-3′	18	151	58	Umeshappa et al. (2010)
	R: 5′-ACGCTCTCCACACACATGAC-3′	20			
Caspase-3	F: 5′-TCTTCAGAGGGGACTGTTGC-3′	20	206	58	Umeshappa et al. (2010)
	R: 5′-ACTTTGAGTTTCGCCAGGAA-3′	20			

### 2.13 Quantification of BTV-24 genome using real-time PCR

The BT viral load in blood and tissues (skin, tongue, thymus, tonsil, trapezius muscle, spleen, PSLN, lungs, heart, and pulmonary artery) at specified time points was quantified by TaqMan probe-based real-time PCR using the specific BTV NS3 (segment-10) probe [(6-FAM)-ARG CTG CAT TCG CAT CGT ACG C-(Tamra-Q)], forward primer: 5′-TGG AYA AAG CRA TGT CAA A-3′, and reverse primer: 5′-ACRTCATCACGAAACGCTTC-3′. The reaction was carried out in Stratagene Mx3005P™ Multiplex QPCR using a One-Step RT-PCR kit (QIAGEN GmbH, Hilden, North Rhine-Westphalia, Germany). The reaction mix was prepared with 2 μg of RNA as template combined with 10 pmol each of forward and reverse primers and probe with other components in the reaction mixture according to the manufacturer’s instructions. The cycling conditions were as follows: reverse transcription at 50 °C for 30 min, initial PCR activation at 95 °C for 15 min, template denaturation at 94 °C for 30 s, primer annealing at 56 °C for 30 s, extension at 72 °C for 30 s for a total of 40 cycles, and final extension at 72 °C for 10 min. Individual cycle threshold (Ct) values were determined and real-time PCR amplification was confirmed by the dissociation curve at the end of the reaction. The standard curve was used for the quantification of BTV RNA in the samples.

### 2.14 Statistical analysis

Data were analyzed using the statistical analysis program GraphPad Prism, Version 5.0 and IBM SPSS Statistics 20.0 software. The average cumulative clinical score was calculated for the scoring of clinical signs. The mean of the BTV-infected and uninfected control groups at specified time intervals was calculated and expressed as mean ± standard error. The clinical scores were tested for statistical significance using the Kruskal–Wallis test and Wilcoxon signed-rank test for non-parametric distribution. The differences between the baseline value (taken at day 0) and the values obtained at each time point in the BTV-infected group were analyzed using paired *t*-test. The differences between the uninfected control and BTV-infected groups at the same time point were analyzed using a two-way analysis of variance (ANOVA) with Bonferroni *post-hoc* test. For all comparisons, differences were considered significant at *p* < 0.05. For real-time PCR analysis, the data obtained were analyzed by using the 2^–ΔΔCt^ method, and the relative expression (ΔCt) for each group was statistically analyzed by repeated-measures ANOVA with Bonferroni *post-hoc* test.

## 3 Results

### 3.1 Clinical signs

**Figure 1:**
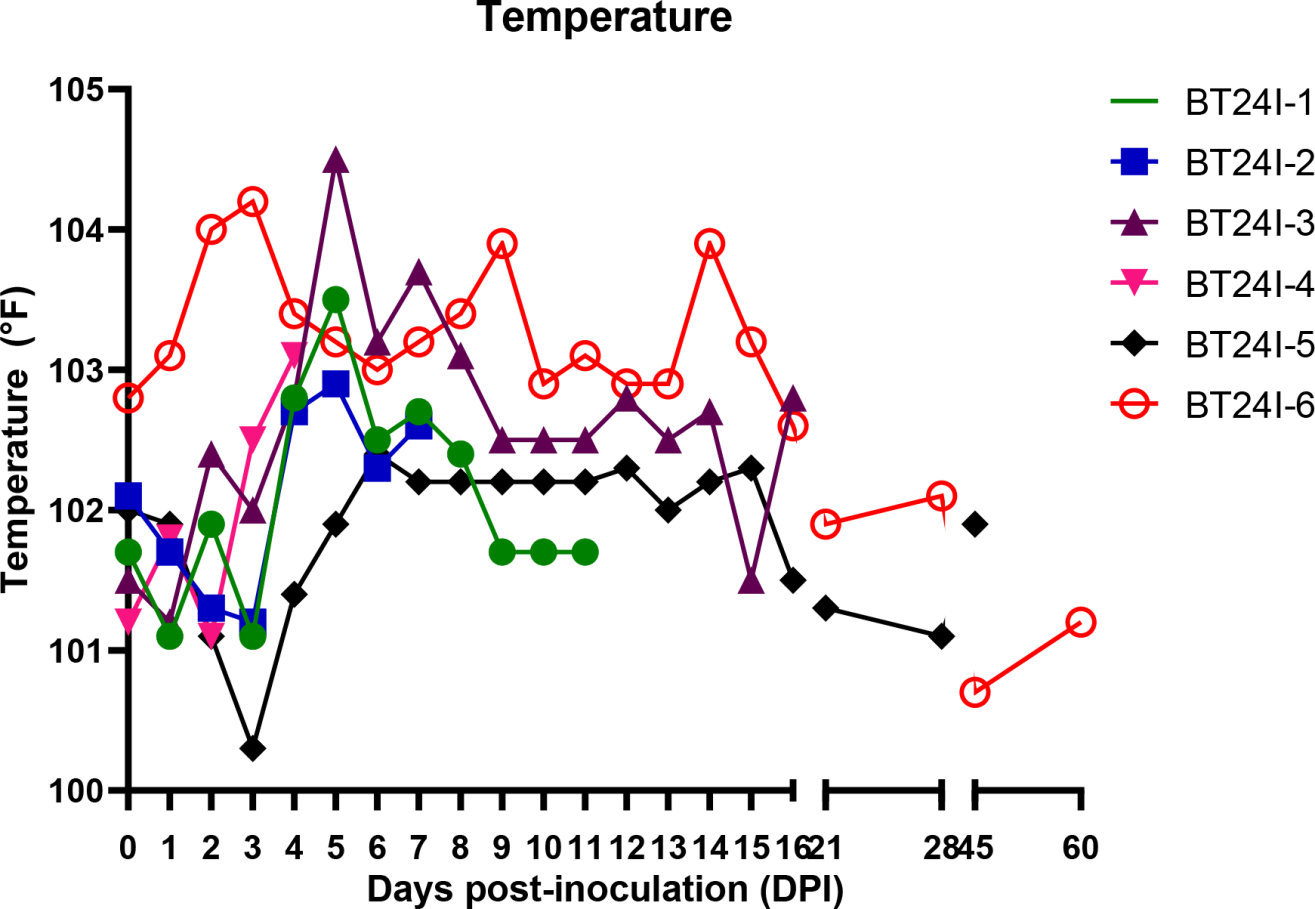
Rectal temperature in bluetongue virus serotype 24-infected animals.

**Figure 2:**
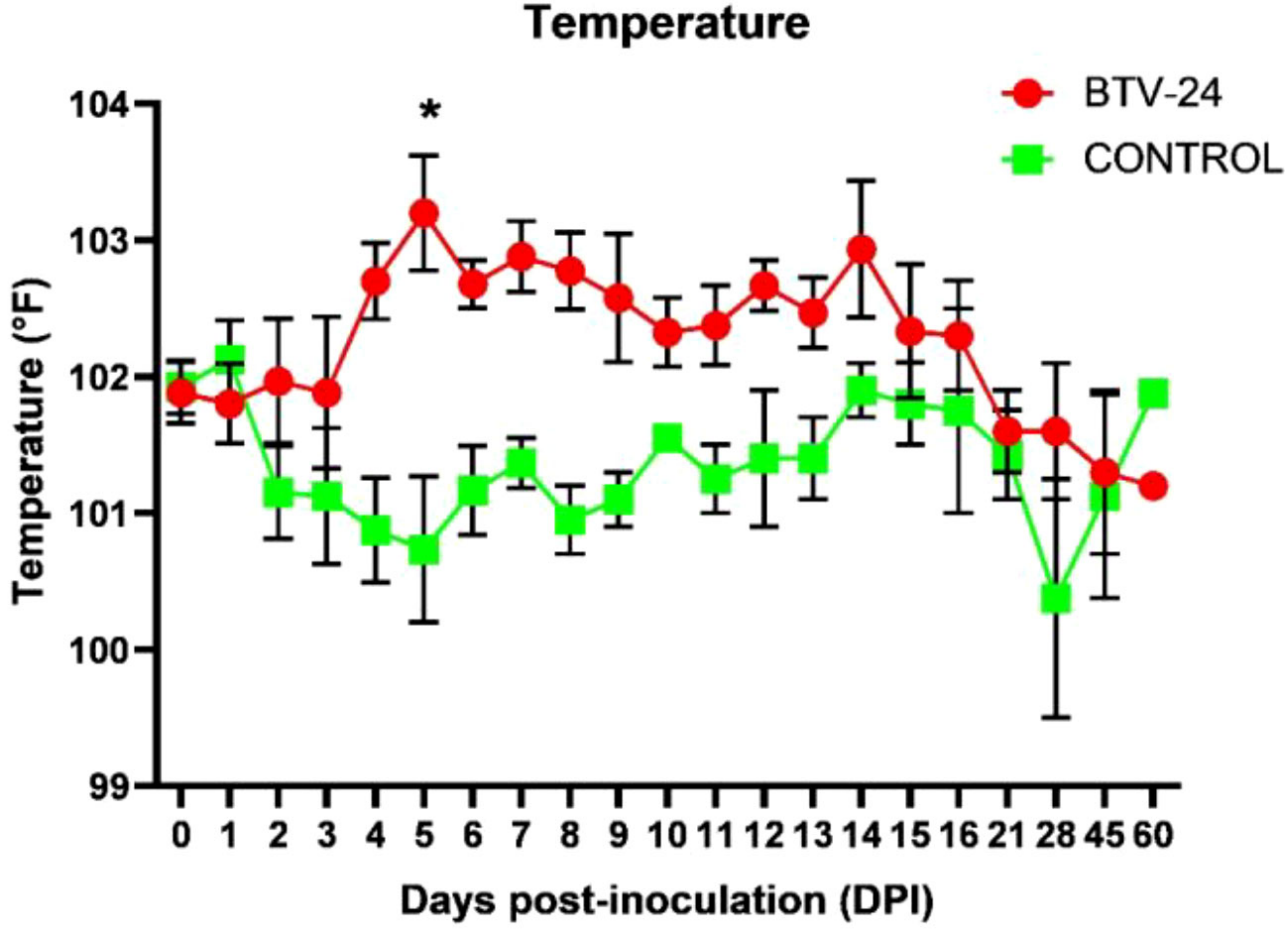
Rectal temperature of BTV-24-infected and uninfected control groups. Results are presented as mean ± SE at each time point. Asterisk (*) indicates a significant difference (*p* < 0.05) between groups. Two-way ANOVA with Bonferroni *post-hoc* test was used.

In BTV-24-infected animals, a significant (*p* < 0.05) increase in rectal temperature was observed at 4 and 5 DPI when compared to uninfected control animals. BTV-infected animals (BT24I-6) developed hyperthermia at 2 and 3 DPI (~40°C/104°F) with a second peak at 9 and 14 DPI. BT24I-3 showed peak pyrexia at 5 (40.3°C/104.5°F) and 6 DPI (39.8°C/103.6°F). Two animals, BT24I-1 and BT24I-4, developed temperatures of 39.5°C/103.1°F and 39.7°C/103.5°F at 4 and 5 DPI, respectively (**Figures 1**, **2**). A significant difference in the clinical sign scores was observed between BTV-24 and uninfected control animals at 4, 6, 7, and 12 DPI (**Supplementary Tables 4–7**). Infected animals showed moderate disease progression with congestion of conjunctival and oral mucus membranes in four out of six animals, cyanosis of the tongue in two out of six animals, and serous to catarrhal nasal discharge in three out of five animals at 5 DPI. During the mid-course of the study (11–15 DPI), clinical signs were mild in severity. Compared to uninfected controls, oral lesion score showed a significant difference at 4 DPI in infected animals. The control animals remained normal throughout the study period.

### 3.2 Gross pathological lesions

**Figure 3:**
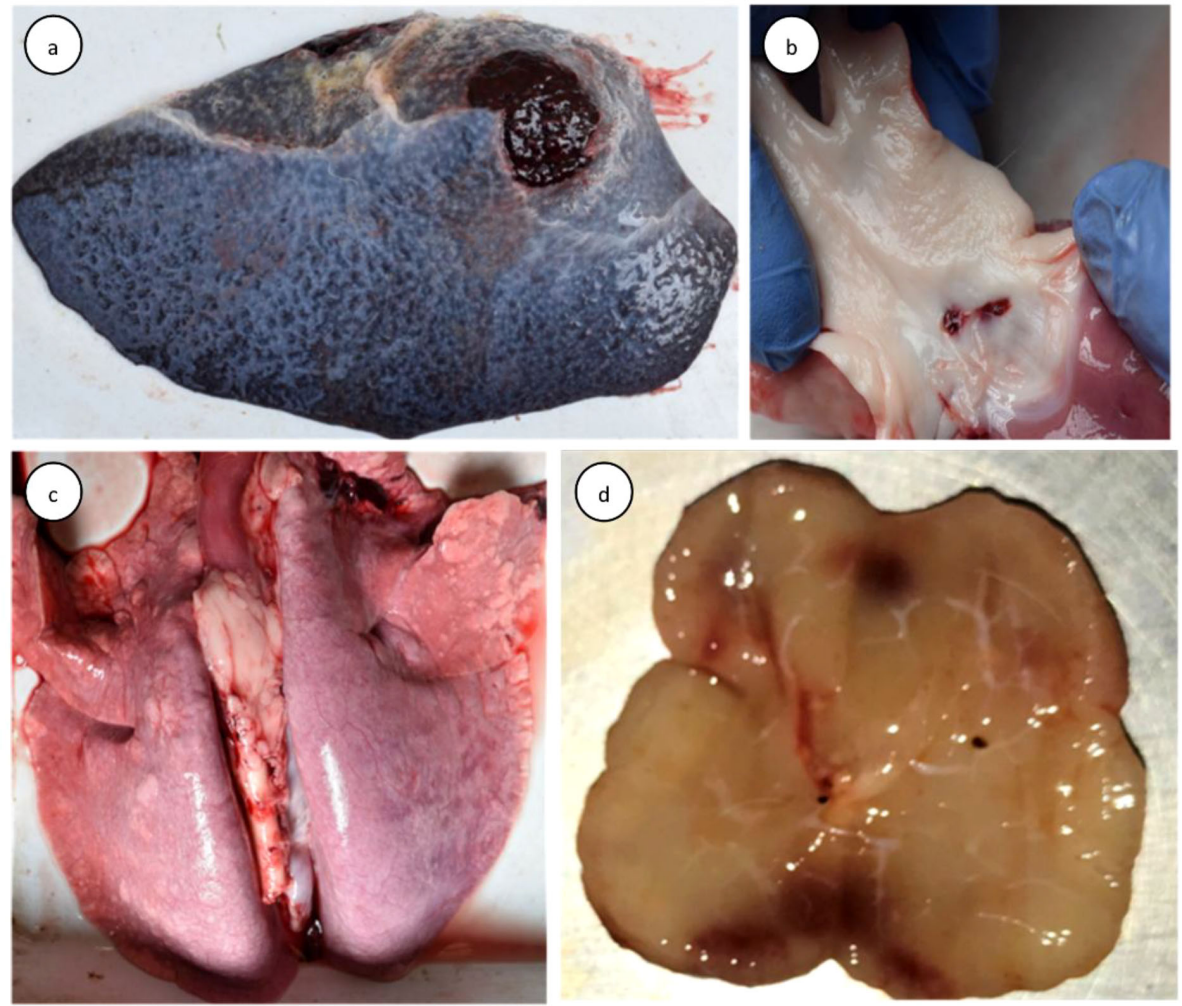
Gross pathological lesions in BTV-24-infected sheep. **(a)** Enlarged spleen at 4 DPI. **(b)** Subintimal hemorrhage on the base of pulmonary artery at 7 DPI. **(c)** Non-collapsed, heavy, and congested lungs at 7 DPI. **(d)** Edematous and enlarged PSLN with cortical hemorrhages at 11 DPI.

The BTV-24-infected animals sacrificed at different time intervals showed varying degrees of gross pathological lesions. Enlarged spleen with prominently reactive splenic white pulp visible on cut sections was observed in four out of six animals. Pulmonary edema and leathery consistency of the lungs were apparent in sheep sacrificed at different time intervals (**Figures 3a, c**). Enlarged (4/6 animals) and hemorrhagic (3/6 animals) prescapular, mesenteric (4/6 animals), and mediastinal (3/6 animals) lymph nodes were observed (**Figure 3d**). Mucosal edema and vascular congestion in the small intestine and ileocecal lymph nodes were observed in three out of six animals sacrificed at 4, 7, and 16 DPI. The pathognomonic lesion of BT, subintimal hemorrhage at the base of the pulmonary artery, was observed in sheep (BT24I-2) sacrificed at 7 DPI (**Figure 3b**). Kidneys showed hemorrhages in medulla. The meningeal blood vessels were congested. The total gross pathological lesions score was higher in BTV-24-infected animals at 7 DPI when compared with the uninfected control animals. The uninfected control animals did not show any gross pathological lesions in the observed organs.

### 3.3 Histopathological lesions

#### 3.3.1 Four days post-inoculation

**Figure 4:**
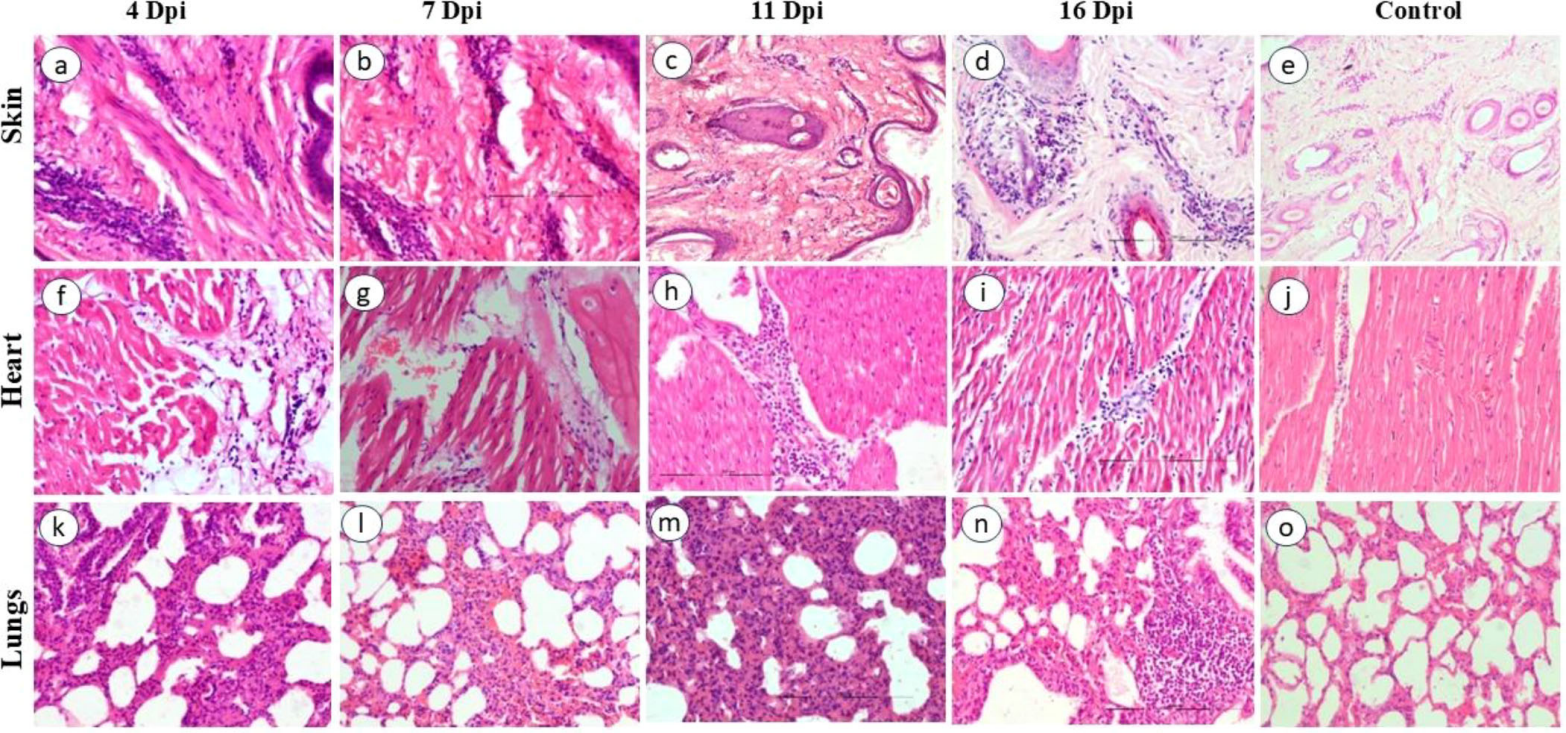
Histopathological lesions in BTV-24-infected sheep at different DPI. **(a)** Dermis of skin showing moderate infiltration of mononuclear inflammatory cells at 4 DPI. H&E ×200. **(b)** Loose connective tissue and severe perivascular infiltration of mononuclear cells at 7 DPI. H&E ×200. **(c)** Fragmented and necrotic hair follicles and edematous fluid surrounding contracted and degenerated follicles at 11 DPI. H&E ×100. **(d)** Infiltration of mononuclear cells around the blood vessels of dermis at 16 DPI. H&E ×200. **(e)** Skin of control animal at 7 DPI. **(f)** Heart showing increased interfascicular connective tissue with edema and mild infiltration of mononuclear inflammatory cells at 4 DPI. H&E ×200. **(g)** Heart showing separation, thinning of muscle fibers, and interstitial infiltration of MNCs at 7 DPI. H&E ×200. **(h)** Heart showing interstitial and perivascular infiltration of MNCs at 11 DPI. H&E ×200. **(i)** Heart showing marked interfascicular infiltration of MNCs at 16 DPI. H&E ×200. **(j)** Heart of control animal at 7 DPI. **(k)** Lungs showing interalveolar septal thickening at 4 DPI. H&E ×200. **(l)** Lungs showing thickening of interalveolar septa and severely engorged pulmonary vessels at 7 DPI. H&E ×200. **(m)** Lungs showing severe interstitial proliferation and thickening of interalveolar septae at 11 DPI. H&E ×200. **(n)** Lungs showing mild thickening of interalveolar septae and mild hyperplasia of BALT at 16 DPI. H&E ×200. **(o)** Lungs of control animal at 7 DPI. H&E ×200.

**Figure 5:**
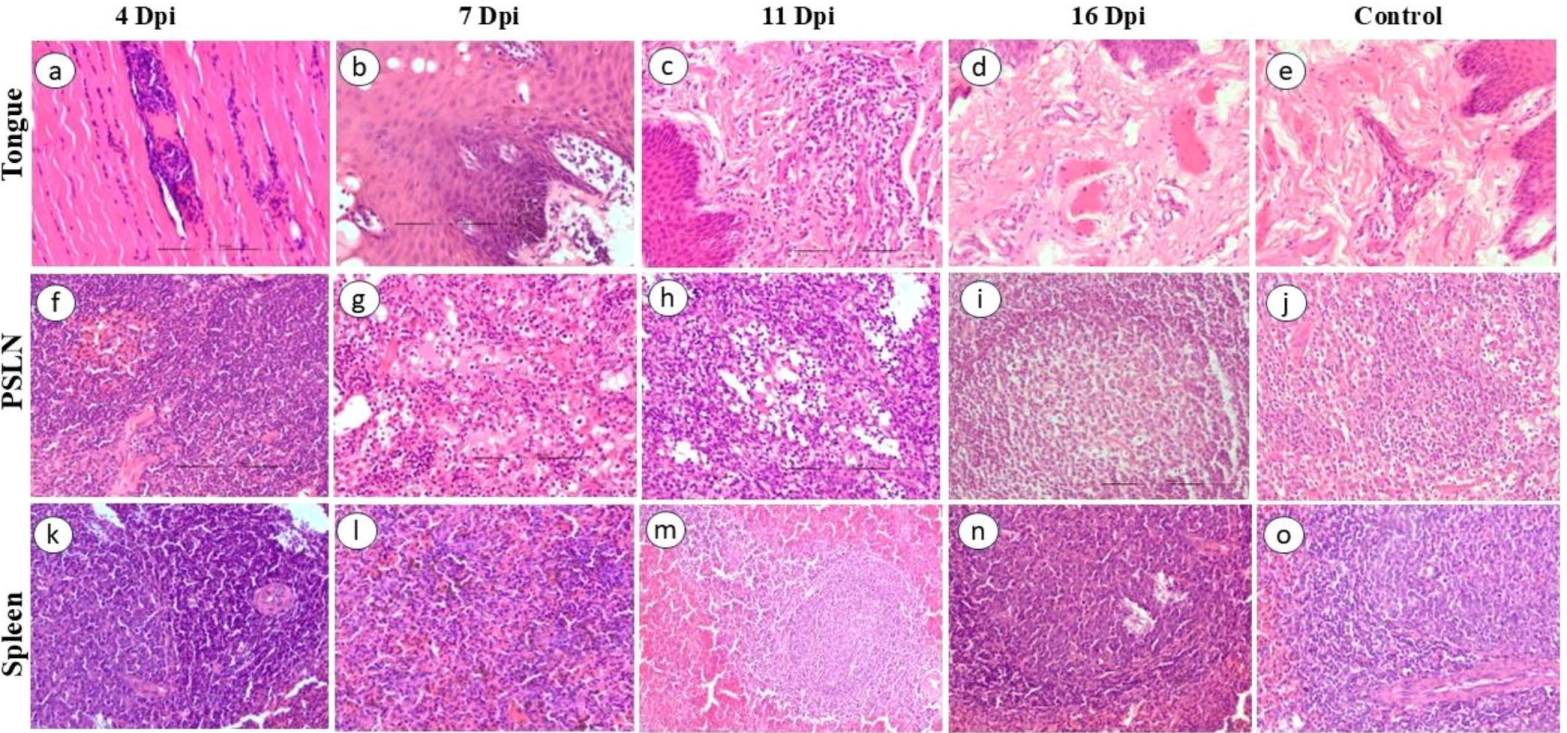
Histopathological lesions in BTV-24-infected sheep at different DPI. **(a)** Tongue musculature showing hyaline degeneration, loss of striations, and mononuclear cell (MNC) infiltration at 4 DPI. H&E ×200. **(b)** Epithelium of tongue showing microvesicle formation with inflammatory cell infiltration and epidermal hyperplasia at 7 DPI. H&E ×200. **(c)** Lamina propria of tongue showing severe perivascular and diffuse infiltration of MNCs at 11 DPI. H&E ×200. **(d)** Mild perivascular infiltration of MNCs in the lamina propria and hyaline degeneration of musculature at 16 DPI. H&E ×200. **(e)** Tongue of uninfected control animal at 7 DPI. H&E ×200. **(f)** PSLN showing hemorrhage in the cortex at 4 DPI. H&E ×200. **(g)** PSLN showing severe edema of medullary sinuses at 7 DPI. **(h)** PSLN showing lymphoid depletion with shrunken and pyknotic nuclei of lymphocytes at 11 DPI. H&E ×200. **(i)** Active germinal center of the follicle at 16 DPI. H&E ×200. **(j)** Section of PSLN of uninfected negative control at 7 DPI. H&E ×200. **(k)** Proliferative changes, thickening of tunica media of splenic blood vessels, white pulp hyperactivity, and germinal center formation at 4 DPI. H&E ×200. **(l)** Red pulp congestion and severe hemosiderosis at 7 DPI. H&E ×200. **(m, n)** white pulp hyperactivity and germinal center formation at 11 and 16 DPI. H&E ×200. **(o)** Spleen of uninfected control at 7 DPI.

The skin of the BTV-infected animals showed moderate to severe vascular reactions in the superficial and deep dermis along with mild edema and mononuclear cell (MNC) infiltration (**Figure 4a**). Focal and mild to moderate infiltration of MNCs, edema, and fibrinous exudates were observed in the interstitium and around the muscle fibers of heart (**Figure 4f**). Lungs showed congestion of alveolar capillaries and marked interalveolar septal thickening (**Figure 4k**). Severe hemorrhage was observed in the thymic medulla. Mild to moderate infiltration of mononuclear inflammatory cells around the blood vessels with swollen endothelial cells were observed in the tongue. Musculature of tongue showed hyaline degeneration, loss of striations, and MNC infiltration (**Figure 5a**). Cortical hemorrhages were observed in PSLN (**Figure 5f**). In spleen, white pulp hyperactivity, thickening of tunica media of vessels, and germinal center formation was observed (**Figure 5k**). The uninfected control animals did not show any histopathological lesions in the examined organs.

#### 3.3.2 Seven days post-inoculation

Prominent histopathological lesions were observed in the animal sacrificed at 7 DPI. Skin showed swollen endothelial cells of dermal blood vessels and severe perivascular infiltration of MNCs with few neutrophils. Degenerated and contracted hair follicles, clear eosinophilic fluid-filled spaces around hair follicles and dermal blood vessels, and loosening of connective tissue suggestive of edema were observed in the skin (**Figure 4b**). Focal mononuclear inflammatory reaction was observed in the heart (**Figure 4g**). In the lungs, moderate to severe interalveolar septal thickening septa and alveolar capillary congestion were observed (**Figure 4l**). The tongue showed microvesicle formation, epidermal thickening, and mononuclear inflammatory reaction in the lamina propria (**Figure 5b**). In lymph nodes, vascular congestion and medullary edema were prominent, along with an increased presence of MNCs in the medullary sinuses (**Figures 5g, j**). In the cortex, lymphoid follicles were active with germinal center formation, lymphoid depletion, deposition of homogenous pinkish materials, and hemorrhages. Spleen showed proliferative changes with white pulp hyperactivity and germinal center formation, swelling of endothelial cells, red pulp congestion, and severe hemosiderosis (**Figure 5l**). Thickening of the tunica media in trabecular arteries was also noted. Kidneys showed severe vascular congestion, hemorrhages in the medulla, and tubular necrosis. In liver, sinusoidal congestion and periportal infiltration of MNCs were observed. Brain revealed mild gliosis and endothelial swelling (**Figure 5o**). The uninfected control animals did not show any histopathological lesions in the examined organs (**Figures 4e, j, o**).

#### 3.3.3 Eleven days post-inoculation

In the skin, severe vascular damage was observed, especially in the dermis, which showed fragmented and necrotic hair follicles and edematous fluid surrounding contracted and degenerated follicles (**Figure 4c**). In heart, the severity of inflammatory reaction was greater at 11 DPI as compared to other time points. Severe infiltration of MNCs around the blood vessels, in and around necrosed cardiac muscles, and in the epicardium was observed (**Figure 4h**). Proliferation and thickening of interalveolar septae and peribronchial lymphoid hyperplasia were prominent in lungs (**Figure 4m**). Swollen and denuded endothelial cells of blood vessels and severe perivascular infiltration of MNCs were observed in the lamina propria and tunica muscularis of tongue (**Figure 5c**). Active germinal center formation, shrunken and pyknotic nuclei indicating apoptosis of the lymphocytes, and edematous fluid were obvious in the lymph nodes (**Figure 5h**). Proliferative changes of white pulp and germinal center formation was observed in spleen (**Figure 5m**). MNC infiltration was observed in the trapezius muscles. Thymic blood vessels were found to be severely congested. Tracheal mucosa was found to be congested and submucosa showed focal hemorrhages and MNC infiltration. Severe infiltration of MNCs was observed periportally in liver. Kidneys showed vascular congestion and mild to moderate tubular degeneration. Mild to moderate meningeal congestion and infiltration of MNCs were observed in brain. The uninfected control animals did not show any histopathological lesions in the examined organs.

#### 3.3.4 Sixteen days post-inoculation

Mild to moderate infiltration of mononuclear cells was observed around blood vessels of the dermis (**Figure 4d**). Mild to moderate infiltration of MNCs was observed in the heart (**Figure 4i**). Severe thickening of interalveolar septae and focal MNC infiltration were observed in the lungs (**Figure 4n**). Mild perivascular infiltration in the lamina propria and hyaline degeneration of tongue musculature were observed (**Figure 5d**). Lymph node sections showed formation of distinct lymphoid follicles with germinal center formation, spleen sections showed white pulp hyperactivity and germinal centre formation (**Figure 5n**) and reticuloendothelial cell hyperplasia in the mantle zone (**Figure 5i**) Hyaline degeneration and inflammatory reaction were observed in the trapezius muscle. The uninfected control animals did not show any histopathological lesions in the examined organs.

#### 3.3.5 Forty-five days post-inoculation

Mild to moderate infiltration of MNCs was observed in the interstitium of cardiac muscles. Sections of lymph nodes showed lymphoid hyperplasia and germinal center formation. Prominent lymphoid follicles with germinal centers and lymphoid hyperplasia were evident in the spleen. The uninfected control animals did not show any histopathological lesions in the examined organs.

#### 3.3.6 Sixty days post-inoculation

Lymph node sections showed mild hyperplasia of reticuloendothelial cells in mantle zone and mild to moderate lymphocyte proliferation in the white pulp of spleen. The uninfected control animals did not show any histopathological lesions in the examined organs.

### 3.4 Immunohistochemical localization BTV antigen

**Figure 6:**
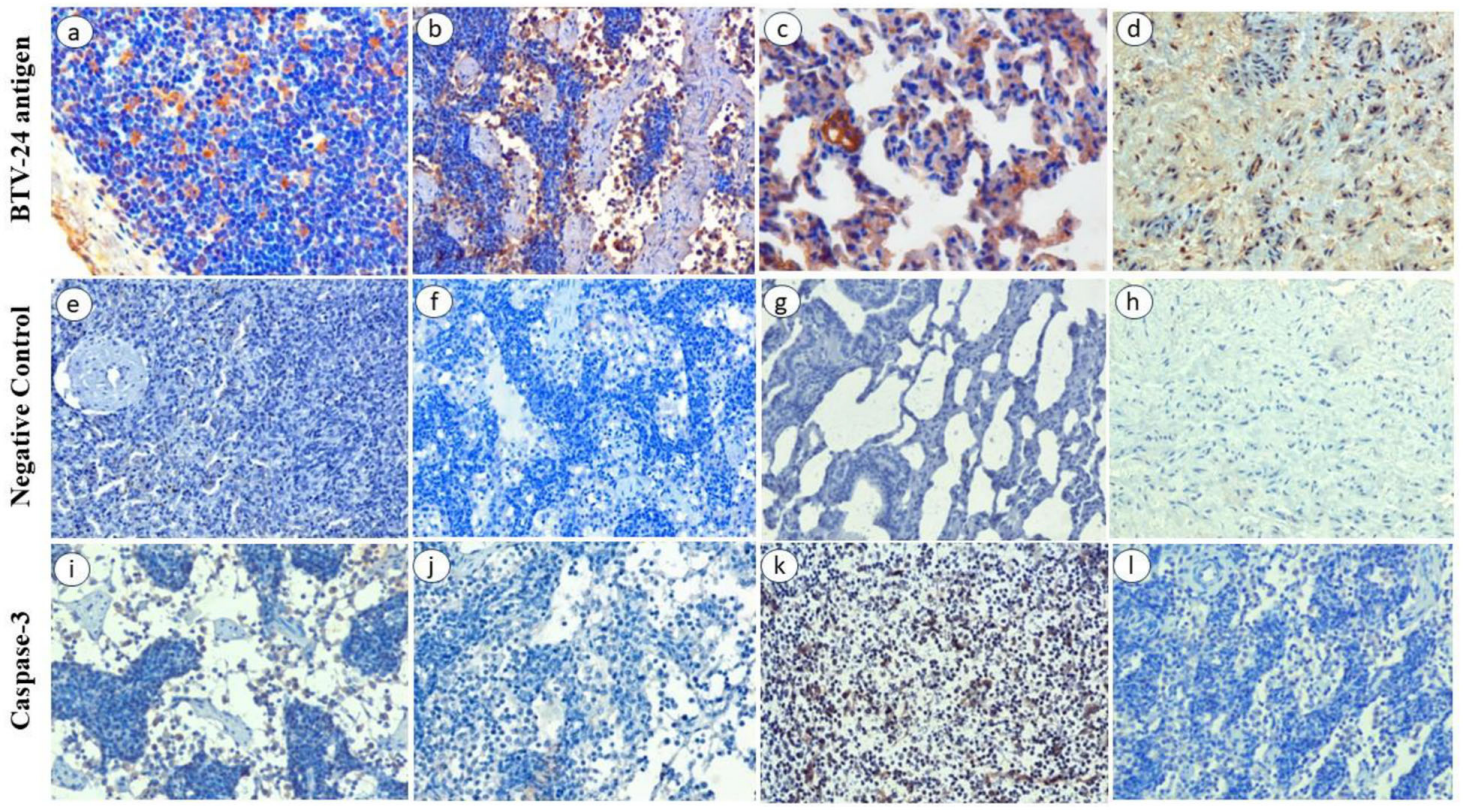
Immunohistochemical localization BTV antigen in BTV-24-infected sheep at different DPI. **(a)** Cytoplasm of macrophages and lymphocytes in PSLN showing BTV-specific signals at 7 DPI. IP-DAB-MH ×200. **(b)** Positive immunolabeling of BTV antigen in the macrophages and mononuclear cells in the medullary sinuses of PSLN at 7 DPI. IP-DAB-MH ×200. **(c)** Positive immunolabeling of BTV antigen in alveolar epithelial cells of lungs at 11 DPI. IP-DAB-MH ×200. **(d)** Positive immunolabeling of BTV antigen in endothelial cells and muscle bundles of tunica media of pulmonary artery at 7 DPI. IP-DAB-MH ×200. **(e, f)** Negative control of PSLN at 7 DPI. **(g)** Negative control of lungs at 7 DPI. **(h)** Negative control did not show positive immunolabeling in the pulmonary artery at 7 DPI. IP-DAB-MH ×200. **(i, k)** Active caspase-3 expression in the cytoplasm and nuclei of mononuclear cells in the medullary sinuses, follicles, and parafollicular area of lymph node at 7 DPI. IP-DAB-MH ×200. **(j, l)** Uninfected negative control at 7 DPI. IP-DAB-MH ×200.

In BTV-24-infected animals, positive immunolabeling of BTV antigen was observed in various organs such as lungs, PSLN, spleen, liver, brain, and tongue from 4 to 11 DPI. Positive immunolabeling of BTV antigen was observed in the cytoplasm of MNC infiltrates in the skin at 4, 7, and 11 DPI. Positive immunolabeling of BTV antigen was observed in the cytoplasm of macrophages and lymphocytes in the cortex and in the macrophages and MNCs in the medullary sinuses of lymph nodes (**Figures 6a, b**). Endothelial cells and muscle bundles of tunica media of pulmonary artery (**Figure 6d**), cytoplasm of bronchiolar and alveolar epithelial cells and mononuclear inflammatory cells of lungs (**Figure 6c**), and splenocytes of white pulp area of spleen showed positive immunolabeling of BTV antigen. In liver, positive signals were observed in the cytoplasm of MNCs infiltrate. Positive immunolabeling of BTV antigen was observed in the neurons and endothelial cells of cerebrum. The uninfected control animals did not show any positive immunoreactivity (**Figures 6e–h**). Scoring of immunohistochemistry signal intensity for BTV antigen in various organs at different DPI is presented in **Table 2**. Furthermore, caspase-3 expression was observed in the parafollicular area as well as in medulla of lymph nodes (**Figures 6i, k**). The uninfected control animals did not show any positive reactivity (**Figures 6j, p**).

**Table 2:** Scoring the intensity of immunohistochemistry signals of BTV antigen in various organs at different DPI.

Organs	Days post-inoculation (DPI)						
	4	7	11	16	21	45	60
Skin	+++	+++	++++	+	+	-	-
Tongue	++	++	+++	+	-	-	-
Muscle	-	-	+	++	-	-	-
PSLN	-	+++	+++	-	-	-	-
Spleen	+	+++	+	+	-	-	-
Thymus	++	-	+	-	-	-	-
Lungs	+	++	+++	+++	-	-	-
Heart	++	+	+++	-	-	-	-
Pulmonary artery	-	++	-	+	-	-	-
Brain	-	-	-	-	-	-	-

-, no immunostaining; +, weak immunostaining; ++, moderate immunostaining; +++, strong immunostaining.

### 3.5 Humoral immune response against BTV-24

**Figure 7:**
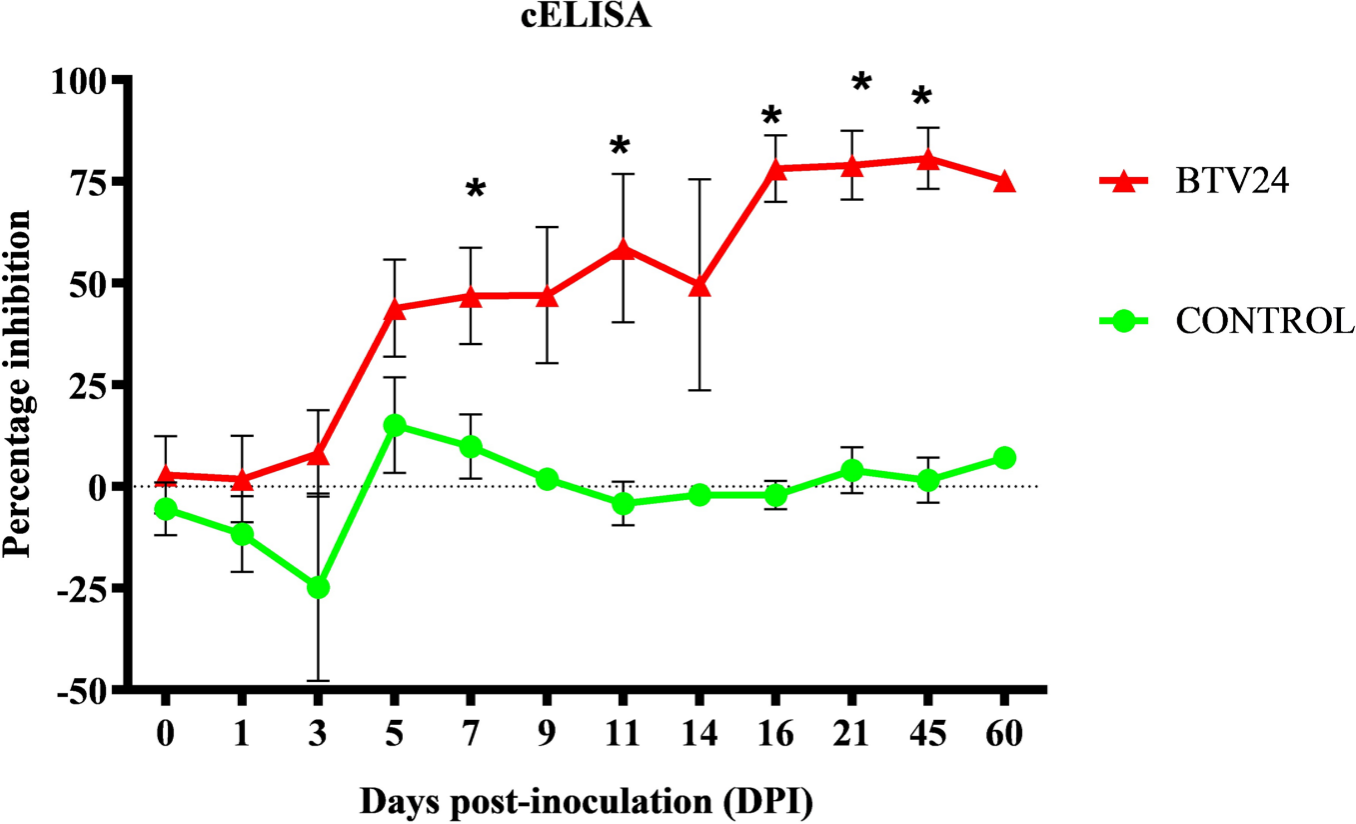
Detection of BTV antibodies by c-ELISA in BTV-24-infected (red line) and uninfected control (green line) animals at different days post-inoculation (DPI). Serum samples with percent inhibition (PI) values ≥ 45% were considered positive. Data are presented as mean ± SE. Asterisk (*) indicates significant difference (*p* < 0.05) between groups.

The humoral immune response was assessed by the development of BTV-specific antibodies. The BTV-24-infected animals remained seronegative until 5 DPI. Thereafter, the PI values exceeded 45% and remained high until the end of the experiment (**Table 3**). A significant increase in antibody titers was observed in BTV-24-infected animals at 5, 11, 16, 21, 45, and 60 DPI (**Figure 7**).

**Table 3:** Percentage inhibition (antibody titer) values of BTV-24-infected and uninfected control groups at different time intervals.

Groups	Days post-inoculation (DPI)											
	0	1	3	5	7	9	11	14	16	21	45	60
BTV-24 infection	2.83 ± 9.49	1.83 ± 10.58	8.10 ± 10.61	43.83 ± 11.91	46.85 ± 11.88*	47.06 ± 16.72	58.64 ± 18.31*	49.55 ± 25.94	78.12 ± 8.22*	79.00 ± 8.43*	80.67 ± 7.49*	75.29 ± 0.00*
Uninfected control	−5.50 ± 6.46	−11.70 ± 9.34	−24.81 ± 23.08	15.08 ± 11.79	9.84 ± 7.91	1.80 ± 2.65	−4.15 ± 5.35	−2.01 ± 0.38	−2.12 ± 3.51	4.02 ± 5.64 ^b^	1.58 ± 5.56	7.13 ± 0.10

−, negative for BTV antibodies; +, positive for BTV antibodies. Results were presented as mean ± SE at each time point. Asterisk (*) indicates significant difference (*p* < 0.05). Two-way ANOVA with Bonferroni *post-hoc* test was used.

### 3.6 Cell-mediated immune responses against BTV-24

#### 3.6.1 Kinetics of CD4^+^ and CD8^+^ T lymphocytes

**Figure 8:**
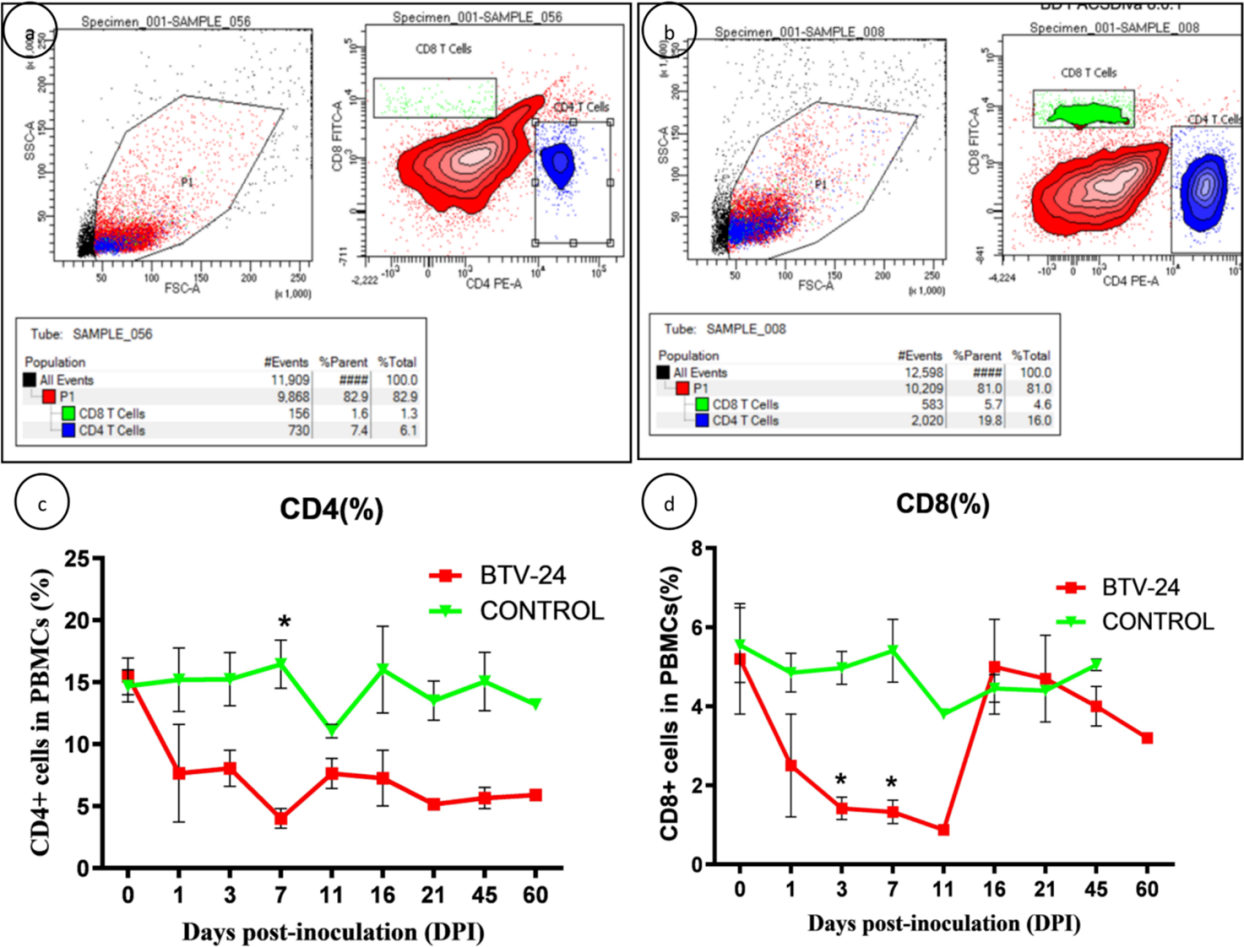
Analysis of kinetics of CD4^+^ and CD8^+^ T lymphocytes. **(a, b)** Dot plot showing flow cytometric analysis of CD4^+^ and CD8^+^ T lymphocytes after staining with PE and FITC, respectively, in the PBMCs of BTV-24-infected **(a)** and uninfected control **(b)** groups at 7 dpi. Lower right quadrant (FL2-H) represents CD4^+^ T lymphocytes (PE), while upper left quadrant (FLI-H) shows CD8^+^ T lymphocytes (FITC). **(c, d)** Line diagram showing the percentage of CD4^+^ and CD8^+^ T lymphocytes in BTV-24-infected and uninfected control groups. Results are presented as mean ± SE at each time point.

In BTV-24-infected animals, there was a significant decrease of CD4^+^ T lymphocytes at 7 DPI and CD8^+^ T lymphocytes at 3 and 7 DPI. There was an increased CD4^+^/CD8^+^ T lymphocyte ratio at 11 DPI in BTV-24-infected animals. A decrease in CD4^+^ and CD8^+^ T cells was observed in the PSLN with lowest levels at 11 DPI, followed by an increase at 16 DPI. A decrease in the CD4^+^ and CD8^+^ T cells was observed in the spleen of BTV-infected animals at 7 DPI and a further increase of the cell population was observed at 11, 16, and 60 DPI (**Figure 8**).

### 3.7 Bluetongue virus quantification

Absolute quantification of bluetongue virus load was done using the NS3 gene of BTV in blood and various organs (skin, tongue, thymus, tonsil, trapezius muscle, spleen, PSLN, lungs, heart, and pulmonary artery) collected from the BTV-24-infected animals at different time intervals. A standard curve was generated with an efficiency of 101.61% and *R*^2^ = 0.996 (**Supplementary Figure 1**). The copy number of unknown samples was extrapolated from the standard curve. The log_10_ BTV copies per 500 ng of RNA from blood was considered for quantification. In blood, peak viremia was observed from 1 DPI, followed by a second peak at 9 DPI, after which the virus titer declined. A statistically significant increase in viral load was observed at 9 DPI (**Table 4**).

**Table 4:** Quantification of BTV viremia by real-time PCR in blood at different DPI.

Animal number	Days post-inoculation (DPI)											
	1	3	5	7	9	11	14	16	21	28	45	60
BT24I-1	NVD	1.62	3.05	3.60	2.82	2.64	S	S	S	S	S	S
BT24I-2	0.60	NVD	3.45	3.21	S	S	S	S	S	S	S	S
BT24I-3	NVD	2.39	2.55	2.64	2.63	2.75	NVD	NVD	S	S	S	S
BT24I-4	NVD	1.40	2.22	S	S	S	S	S	S	S	S	S
BT24I-5	NVD	1.54	1.22	3.10	2.17	3.00	NVD	1.57	3.61	3.70	NVD	NVD
BT24I-6	NVD	1.87	NVD	0.71	2.24	1.14	1.60	NVD	NVD	NVD	NVD	NVD

NVD, no viremia detected; S, sacrificed. Virus load in blood was depicted as log_10_ BTV copies/500 ng RNA.

The bluetongue virus load was quantified in skin, tongue, thymus, tonsil, trapezius muscle, spleen, PSLN, lungs, heart, and pulmonary artery samples. In the BTV-24-infected group, consistently higher viral copy numbers were detected in the skin, tongue, PSLN, heart, and pulmonary artery. The highest viral load was recorded in the pulmonary artery of the animal sacrificed at 16 DPI. The highest viral load was recorded in skin and tongue at 4 DPI, in skin and heart at 7 DPI, in tongue and heart at 11 DPI, and in tonsil, PSLN, and tongue at 16 DPI (**Table 5**).

**Table 5:** Quantification of BTV viremia by real-time PCR in various organs at different DPI.

Organs	Days post-inoculation (DPI)					
	4	7	11	16	45	60
Skin	2.59	2.36	2.39	1.70	NVD	NVD
Tongue	2.53	1.95	3.33	2.40	NVD	NVD
Thymus	NVD	NVD	1.83	1.55	NVD	NVD
Tonsil	2.36	NVD	NVD	2.76	NVD	NVD
Trapezius muscle	2.54	1.94	NVD	2.25	NVD	NVD
Spleen	1.67	0.66	NVD	2.34	NVD	NVD
PSLN	1.81	1.50	0.84	2.65	NVD	NVD
Lungs	1.27	0.94	NVD	2.44	NVD	NVD
Heart	2.45	2.11	2.78	2.22	NVD	NVD
Pulmonary artery	2.24	1.58	0.63	4.42	NVD	NVD

NVD, no viremia detected. Virus load in various organs was depicted as log_10_ BTV copies/500 ng tissue RNA.

#### 3.7.1 Expression of cytokine genes

**Figure 9:**
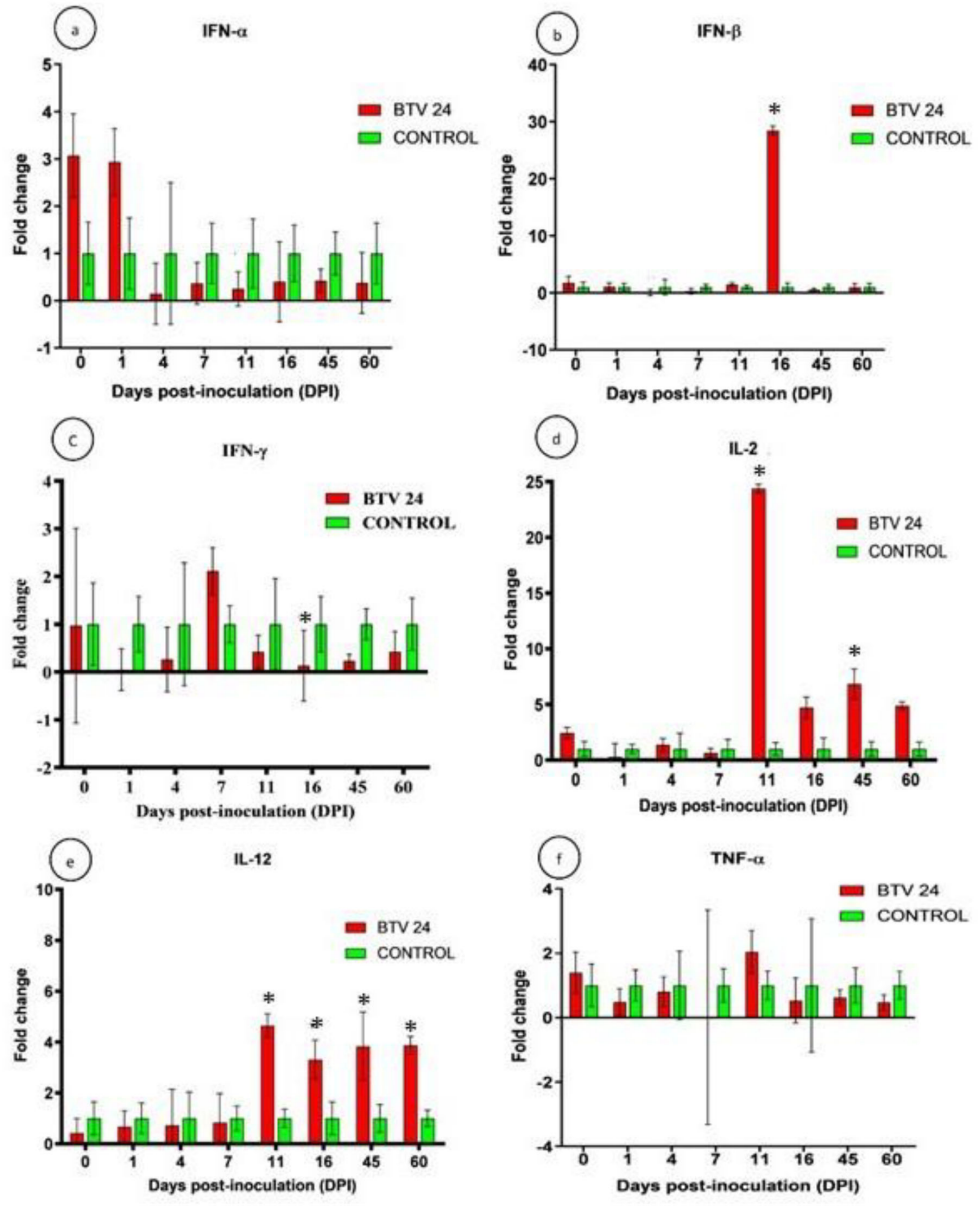
Quantification of cytokine genes’ [IFN-α **(a)**, IFN-β **(b)**, IFN-γ **(c)**, IL-2 **(d)**, IL-12 **(e)**, and TNF-α **(f)**] expressions in PBMCs by qRT-PCR in BTV-24-infected (red bars) and uninfected control (green bars) groups at various time points. Results were presented as bar diagram with mean ± SE at each time point.

**Figure 10:**
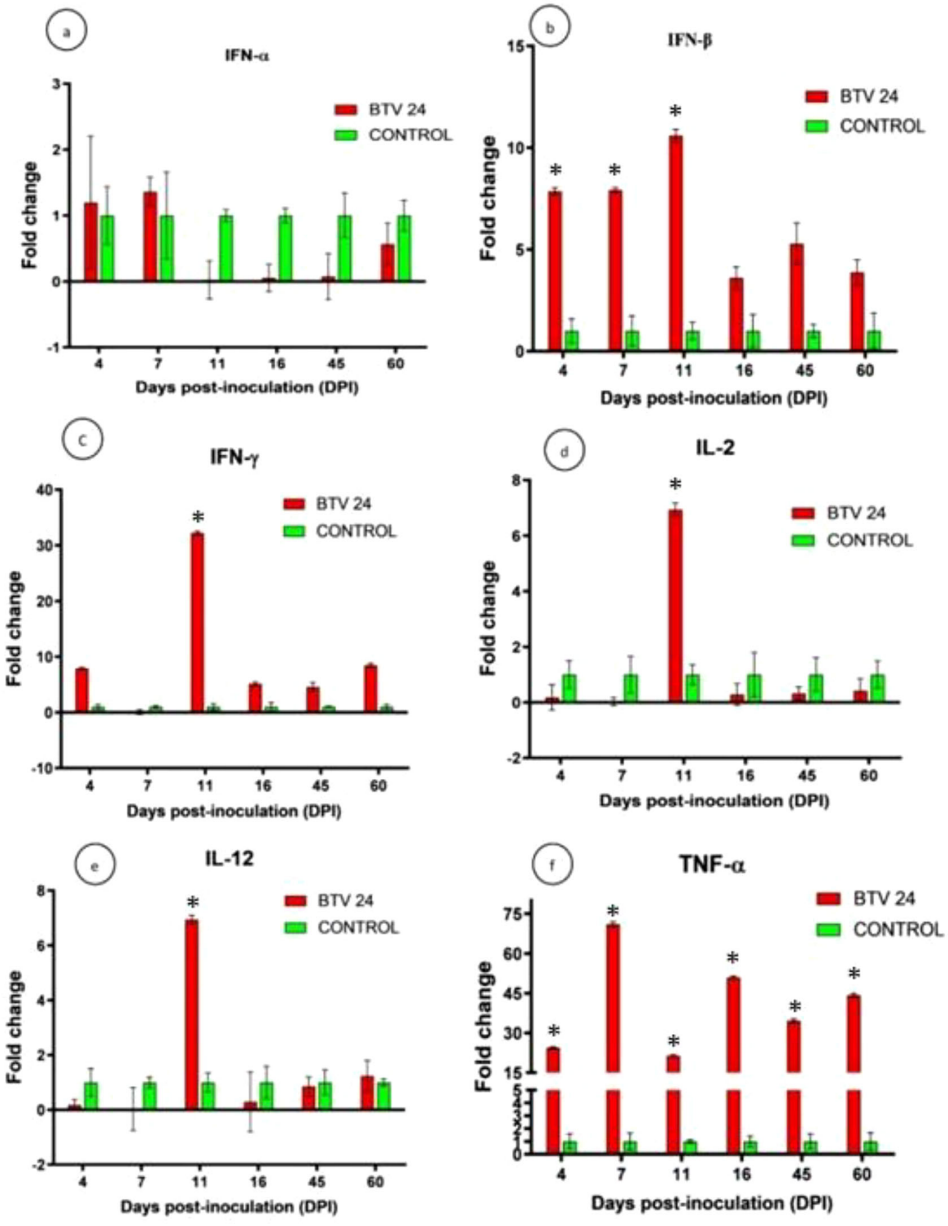
Quantification of cytokine genes’ [IFN-α **(a)**, IFN-β **(b)**, IFN-γ **(c)**, IL-2 **(d)**, IL-12 **(e)**, and TNF-α **(f)**] expressions in spleen by qRT-PCR in BTV-24-infected (red bars) and uninfected control (green bars) groups at various time points. Results were presented as bar diagram with mean ± SE at each time point.

**Figure 11:**
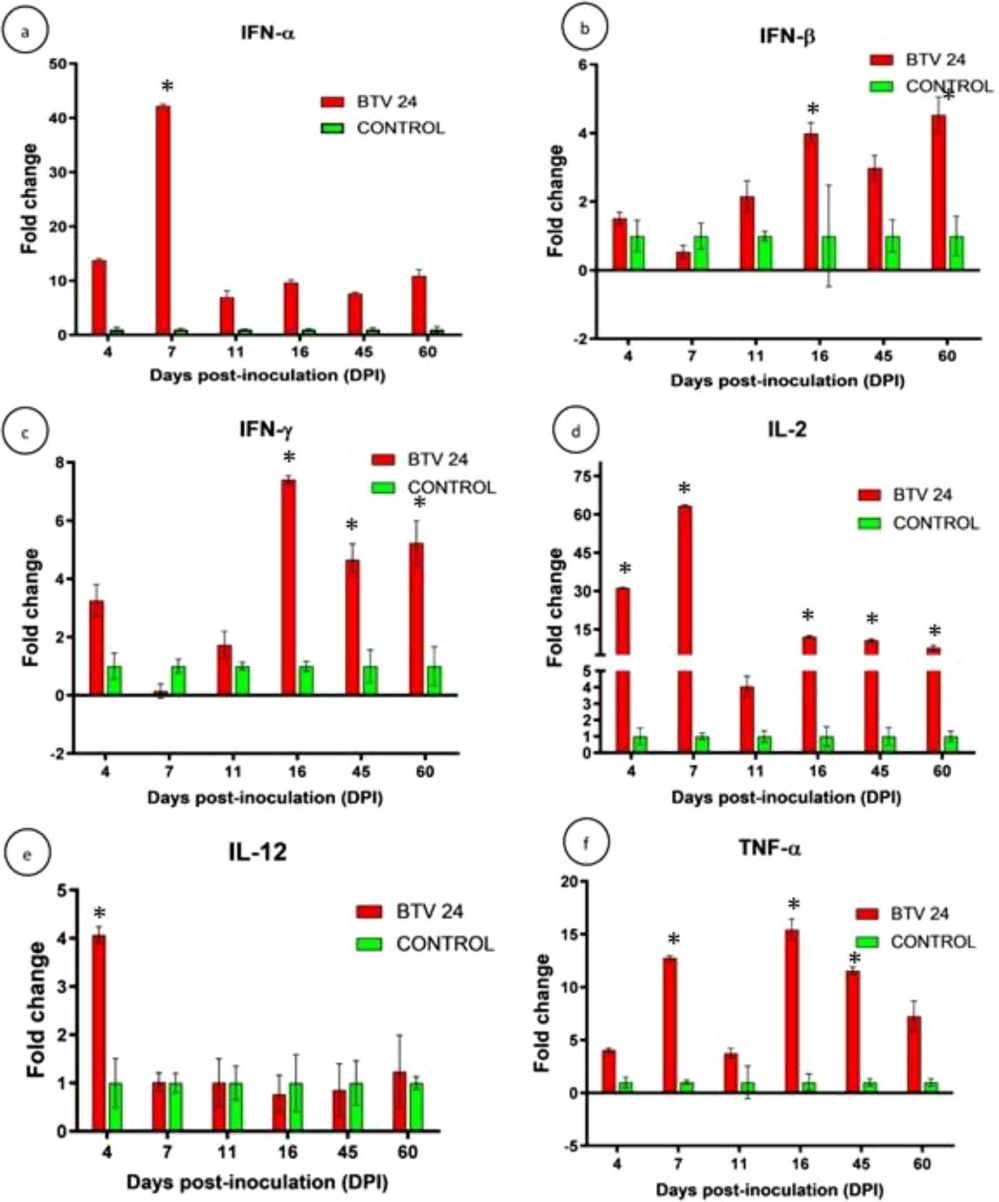
Quantification of cytokine genes’ [IFN-α **(a)**, IFN-β **(b)**, IFN-γ **(c)**, IL-2 **(d)**, IL-12 **(e)**, and TNF-α **(f)**] expressions in PSLN node by qRT-PCR in BTV-24-infected (red bars) and uninfected control (green bars) groups at various time points. Results were presented as bar diagram with mean ± SE at each time point.

**Figure 12:**
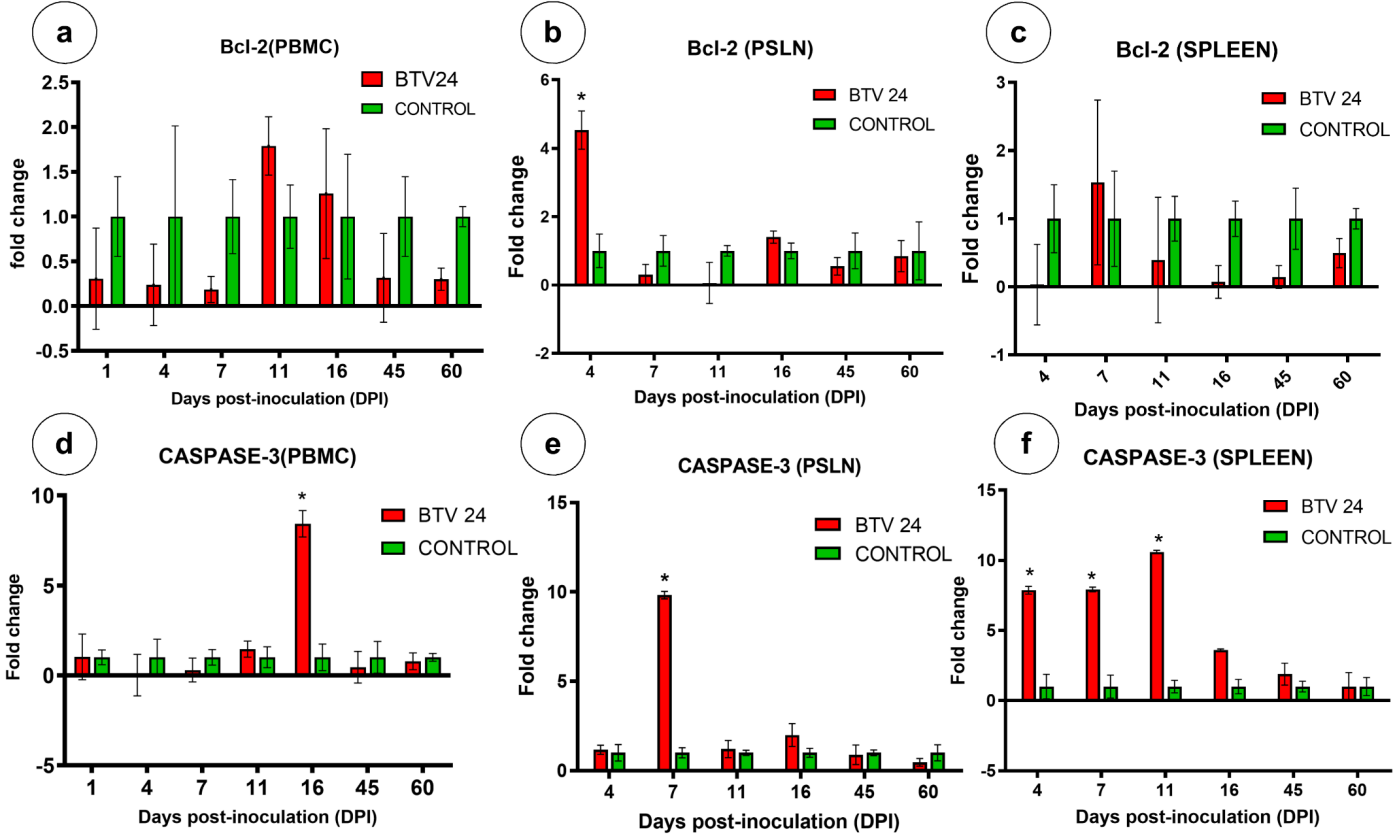
Quantification of apoptotic genes (Bcl-2 and Caspase-3) expressions in PBMCs, PSLN, and spleen by qRT-PCR in BTV-24-infected (red bars) and uninfected control (green bars) groups at various time points. BCl-2 - PBMCs **(a)**, BCl-2 - PSLN **(b)**, BCl-2 - Spleen **(c)**, Caspase-3 - PBMCs **(d)**, Caspase-3 - PSLN **(e)**, and Caspase-3 - Spleen **(f)**. Results were presented as bar diagram with mean ± SE at each time point.

The relative expression patterns of different cytokines in the PBMCs of BTV-24-infected and uninfected control groups were quantified using GAPDH as the housekeeping gene (**Figure 9**). The expression kinetics of IFN-α showed no upregulation in the PBMCs of BTV-24-infected animals (**Figure 9a**). Significant downregulation was observed at 7, 11, and 16 DPI. Peak expression of IFN-α mRNA was observed in the spleen (**Figure 10a**) and draining PSLN (**Figure 11a**) at 7 DPI. Significant downregulation of IFN-β in the PBMCs of BTV-24-infected animals was observed at 4 DPI (**Figure 9b**). Significant downregulation of IFN-β mRNA was observed in the PBMCs of BTV-infected animals at 4 DPI. Peak expression of IFN-β mRNA was observed in the spleen of BTV-infected animals at 11 DPI (**Figure 10b**), whereas the expression was highest at 16 DPI in the draining PSLN (**Figure 11b**) of BTV-infected animals. Initial downregulation of IFN-γ mRNA followed by significant upregulation in the PBMCs of BTV-24-infected animals at 7 and 16 DPI (**Figure 9c**), and the peak expression in the spleen (**Figure 10c**) and draining PSLN (**Figure 11c**) was observed at 11 and 16 DPI, respectively. Following infection, expression of interleukin-2 (IL-2) mRNA in PBMCs showed significant upregulation at 11 DPI (**Figure 9d**) and peak expression of IL-2 mRNA was observed in the spleen (**Figure 10d**) and draining PSLN (**Figure 11d**) at 7 DPI. The fold change in the expression of IL-12 mRNA in PBMCs registered significant up-regulation at 11 to 16 DPI (**Figure 9e**) and up-regulation in the spleen at 4 DPI (**Figure 10e**) and in the draining PSLN (**Figure 11e**) at 7 DPI in the BTV-infected group. Peak expression of TNF-α mRNA was observed at 11 DPI in PBMCs (**Figure 9f**), at 7 DPI in the spleen (**Figure 10f**), and at 7 and 16 DPI in the draining PSLN (**Figure 11f**) of BTV-infected animals. Peak expression of Bcl-2 was observed at 7 DPI in the spleen and 4 DPI in the draining PSLN of BTV-24-infected animals. Peak expression of caspase-3 mRNA was observed in PSLN and spleen sacrificed at 7 and 11 DPI, respectively (**Figure 12**).

## 4 Discussion

Virulence characteristics of various serotypes of bluetongue virus vary significantly, even within the same serotype due to the considerable genetic variability results from mutations in the different genome segments during reassortment (Shaw et al., 2013; Saminathan et al., 2020). There is a shortage of *in vivo* studies to determine the differences in pathogenetic potential of various BTV serotypes in the susceptible host sheep. Furthermore, detailed investigations to elucidate the pathogenetic mechanisms of BTV-24 under experimental conditions in sheep are not available. The present study was the first report on the pathogenesis of serotype BTV-24 in the natural host sheep.

In the present study, animals infected with BTV-24 showed clinical signs of pyrexia, conjunctival and oral mucosal congestion, nasal discharge, and peak viremia at 7 DPI. The observations of the present study were in line with those reported previously under experimental conditions (Umeshappa et al., 2011; Sanchez-Cordon et al., 2013). Serous to catarrhal nasal discharge and cyanosis of the tongue observed in the present study were in concurrence with earlier reports (Barratt-Boyes and MacLachlan, 1994; Umeshappa et al., 2011; Channappanavar et al., 2012; Sanchez-Cordon et al., 2013).

In the present study, most classical and moderate gross pathological lesions were observed in BTV-24-infected animals. The gross pathological lesions observed were akin to earlier reports (Hamblin et al., 1998; Worwa et al., 2010; Umeshappa et al., 2011; Channappanavar et al., 2012; Bianchi et al., 2017). More severe lesions characteristic of fulminant BT such as erosions and ulcerations of oral and gastrointestinal mucosa and endocardial/epicardial hemorrhages reported by earlier studies were not observed in the present study, which might be due to the difference in pathogenesis and/or virulence of the serotype when compared with other studies. Pericardial and pleural effusions observed in the present study in BTV-24-infected animals indicated the widespread endothelial injury leading to vascular leakage (MacLachlan et al., 2008). In the present study, BTV-24-infected sheep showed subintimal hemorrhage at the base of pulmonary artery, which is the pathognomonic lesion of bluetongue. Hemorrhages at the base of the pulmonary artery is considered as infrequent but specific to BTV infection (Antoniassi et al., 2010; Lima et al., 2016). The aforesaid pathological lesions and severe clinical signs in the BTV-24-infected animals indicated the increased virulence of the serotype. In the present study, higher gross pathological lesion scores in BTV-24 infection were correlated with the development of pyrexia and other clinical signs around 4 to 7 DPI.

In the present study, marked histopathological lesions were observed in the BTV-24-infected animals. Lymphoid depletion was observed at 11 DPI, which might be due to the peak levels of IFN-α and the consequent induction of apoptosis in the lymph nodes (Umeshappa et al., 2010). Marked lymphoid hyperplasia was observed in spleen, and these findings were in agreement with previous reports of Umeshappa et al. (2011) and Channappanavar et al. (2012). In the present study, severe hemosiderosis was observed in the spleen of BTV-24-infected animals at 7 DPI, which indicated the hemorrhages due to endothelial damage and/or hemolytic anemia in the infected animals (Saminathan et al., 2021). Hyperplasia of the lymphoid follicle and germinal center formation might be due to the antigenic stimulation by BTV (Saminathan et al., 2021). The edema and fibrin deposition indicated the inflammatory process associated with the infection. In the present study, involvement of MNCs, possibly dendritic cells and sinus macrophages, in the lymph nodes and spleen indicated their role in the pathogenesis of BTV as antigen-presenting cells transporting the antigen from the site of inoculation to the draining lymph nodes, which were earlier documented (Hemati et al., 2009; Saminathan et al., 2020). MNC infiltration in the hyalinized and necrotic trapezius and cardiac muscle fibers was in agreement with the previous findings (Maclachlan et al., 2009; Umeshappa et al., 2011; Worwa et al., 2010; Channappanavar et al., 2012; Bianchi et al., 2017). In the present study, subcutaneous edema and perivascular aggregates of lymphocytes and macrophages were observed in the skin and tongue, which were consistent lesions in BTV-infected animals. In most of the BTV-24-infected animals, endothelial cells’ hypertrophy with or without necrosis was observed, which was in agreement with the findings of Bianchi et al. (2017) and Umeshappa et al. (2011). In the present study, hemorrhages and hyalinization of tunica media of the pulmonary artery in BTV-24-infected animals at 7 DPI might be due to the increased virulence of BTV-24.

BTV antigens could be demonstrated in the germinal center of the lymphoid follicle, subcapsular sinuses, and in the MNCs, presumptively the dendritic cells and macrophages of the medullary region at 7 DPI. In the present study, localization of the antigen in the lymph nodes was in agreement with the observations of Sánchez-Cordón et al. (2010). In contrast, Melzi et al. (2016) studied the sequential pathogenesis of BTV-8 in the lymph nodes of experimentally infected sheep and did not observe any evidence of substantial infection of dendritic cells in the lymph nodes. In spleen, localization of virus was observed in the red pulp and not associated with the follicles of white pulp. Similar observations were made by Sánchez-Cordón et al. (2010) in the spleen. BTV antigen was localized in macrophages and lymphocytes located in peripheral areas of lymphoid follicles, where T cells are found with fewer cells in the inner part of the follicles, which suggested that T cells are more susceptible to BTV infection than B cells (MacLaclan et al., 1990). In the present study, BTV antigen was localized in the bronchiolar lining cells, microvasculature, and inflammatory cells in the alveolar interstitium, which were in line with the observations of Sánchez-Cordón et al. (2010) in sheep.

Slower but stronger antibody response in the BTV-24-infected group indicated an adequate humoral immune response attributed to the stronger antigenic stimulation. However, decreased antibody titers at 9 and 14 DPI might be due to the variation in the individual susceptibility and immune responses. In the present study, mild depletion of lymphoid cells in the lymph nodes was observed at 11 DPI, which might be the reason for the fall of antibody levels from 9 to 11 DPI. One BTV-24-infected animal remained viremic at 21 and 28 DPI, which had high PI values (87% and 88% at 21 and 28 DPI, respectively). Similar observations were reported on bluetongue virus co-circulation with neutralizing antibodies at 23 to 26 DPI in calves (Barratt-Boyes and Maclachlan, 1995). Previous studies on the experimental infection of sheep with BTV-23 demonstrated increased antibody titers from 7 DPI onwards and a peak surge at 13 and 20 DPI, which were correlated with the histopathological lesions such as hyperplastic germinal centers with blastogenesis in the B-cell areas of spleen and lymph nodes (Umeshappa et al., 2011). In the present study, hyperplasia of the germinal centers was evident in the BTV-24-infected animals from 4 DPI onwards.

The cell-mediated immune response likely limits the viral spread during the initial stages of BTV infection in ruminants, although such responses do not lead to the rapid elimination of the virus (Saminathan et al., 2020). Studies revealed that conventional dendritic cells (cDCs) play a critical role in the induction of BTV-specific CD4^+^ and CD8^+^ T-cell proliferation to overcome the BTV infection by synthesis of cytokines, suggesting the importance of cell-mediated immunity (CMI) response (Hemati et al., 2009; Clifton et al., 2010). The immune cell kinetics of CD4^+^ and CD8^+^ T lymphocytes showed a sharp decline in the population of both cells. The CD4^+^ T lymphocytes help to promote B cells’ antibody production required for the generation of cytotoxic and memory CD8^+^ T cells (Chauhan et al., 2024). An increase in the number of CD8^+^ T cells towards normal levels might be possibly due to the generation of cytotoxic T cells during the later stages of infection in BTV-24-infected animals. This was well correlated with the virus clearance and absence of clinical signs and pathological lesions. In the present study, a decrease in CD4^+^ and CD8^+^ T lymphocytes during the early stages of infection was also reported in previous studies (Saminathan et al., 2021). Umeshappa et al. (2010) reported an initial proliferation of CD4^+^ T cells followed by a decline from 7 to 15 DPI and CD8^+^ T-cell proliferation from 7 to 15 DPI.

Type I interferons including IFN-α and IFN-β are involved in cell-mediated immune responses and constitute the first line of defense against viral infections (Saminathan et al., 2019). However, in the present study, a dysregulated expression of Type I interferons was observed, which might have contributed to the observed cellular infection and pathology. An upregulated expression of IFN-α was observed in PBMCs, spleen, and draining lymph nodes from 4 to 7 DPI, which was correlated with viremia (Umeshappa et al., 2011; Saminathan et al., 2019). During viremia, majority of the host cells including MNCs of blood and spleen get infected with BTV, resulting in enhanced production of IFN-α and the subsequent destruction of virally infected cells. The upregulated expression of IFN-β in the BTV-infected animals indicated the induction of an antiviral state in the BTV-24-infected animals at the later stage of infection, indicating a delayed Type I IFN response. The late IFN-β induction indicates a secondary immune response against viral infection. The temporal mismatch between suppressed IFN-α and delayed IFN-β may therefore create an immune window that favors viral amplification during the early phase and contributes to inflammation during the later course of infection as observed in the BTV-24-infected animals as viremia is well correlated with the downregulation of IFN-β expression. These observed findings were verified by severe fever and thrombocytopenia syndrome caused by bluetongue virus and Bunyavirus, which had a strong association with suppressed IFN-β production (Song et al., 2017; Saminathan et al., 2019). The peak level of IFN-α was correlated with the induction of apoptosis of the virally infected cells, triggering potent pro-inflammatory cytokines in the present study as demonstrated by the upregulated expression of apoptotic markers and the induction of weak antiviral cytotoxic immunity during the early stages of infection (Takaoka et al., 2003; Saminathan et al., 2019).

In the present study, a pleiotropic pro-inflammatory cytokine, IFN-γ, which represents adaptive T-cell functions and is associated with specific cellular immunity by NK-cell and cytotoxic T-cell activation against viral diseases, was significantly downregulated in BTV-24-infected animals at 4 DPI, which might have reduced clearance of infected cells and enhanced the replication of virus in different target organs, thereby facilitating virus spread. In the present study, the level of IL-2 in the PBMCs, spleen, and lymph node coincided with the peak viremia observed at 7 and 11 DPI as IL-2 is induced as a result of BTV-induced endothelial damage (Saminathan et al., 2021), indicating that inflammation may be driven more by accumulating viral damage than by protective antiviral activity. The consistently low level of expression of the cytokine IL-12 during the early stages of infection in BTV-24-infected animals might have favored viral replication and spreading by impaired Th1 differentiation, further compromising antiviral CMI (Sánchez-Cordón et al., 2015).

TNF-α is recognized as a proinflammatory cytokine released by macrophages and antigen-specific CTLs while encountering the antigen (Kristensen et al., 2004). Increased expression of TNF-α cytokine in spleen and lymph nodes of the BTV-infected groups indicated the immune response against infection in the target organs, which might be by direct damage and/or apoptosis of the cells in the infected organs. The combined pattern of IFN-α suppression, delayed IFN-β induction, reduced IFN-γ, and dysregulated IL-2/IL-12/TNF-α responses suggests that BTV-24 modulates both innate and adaptive immune pathway and the cytokine imbalance likely promotes early viral replication and immune response, and contributes to the vascular and inflammatory pathology characteristic of severe BT disease.

Increased apoptosis in the spleen and lymph nodes at peak viremia might be the response of the host to ward off virus replication or due to the release of potent cytokines upon antigenic stimulation (DeMaula et al., 2001). Caspase-3 acts as a final effector in all apoptotic pathways, and hence, it is considered as a good marker for apoptosis (Hengartner, 2000). In the present study, upregulated expressions of Bcl-2 and caspase-3 were observed at the later stage of the disease. The present observations of apoptotic signals and mRNA expression profiles of apoptotic markers in the spleen and lymph nodes agreed with the findings of Umeshappa et al. (2010), who reported that a considerable proportion of apoptosis happens in peak viremia, whereas in PBMCs, a higher mRNA expression could be observed in the later stages of the disease and was not related to viremia. The peak level of IFN-α correlated with the upregulated expression of apoptotic markers Bcl-2 and caspase-3 in the spleen and lymph nodes, whereas in PBMCs, an early upregulation of IFN-α mRNA expression was observed, which was correlated with peak viremia.

## 5 Conclusion

The present study reports, for the first time, the pathogenesis of BTV serotype 24 in the natural host sheep. The present study demonstrated a moderate progression of the clinical signs and pathological lesions in BTV-24-infected sheep. The clinical signs varied between individual sheep. The most classical lesion, subintimal hemorrhage at the base of the pulmonary artery, was observed at 7 DPI. Histopathological lesions were primarily confined to the lymph nodes, thymus, spleen, skin, tongue, muscle, heart, and lungs. However, a few animals also exhibited lesions in the pulmonary artery, trachea, liver, and kidneys. BTV-24-infected animals showed strong reactive hyperplasia in lymph nodes and spleen during the later stages of infection. Vascular and inflammatory reactions had noticeable inter-individual variation, whereas the humoral immune response (antibody production) was delayed but robust. Bluetongue virus was consistently detected in the skin, tongue, heart, and trapezius muscle of all the BTV-24-infected animals at different time points. The viral load correlated well with the severity of clinical signs and pathological lesions in different organs. In conclusion, the results of this study can be utilized to develop effective prevention and control strategies, including the design of a suitable vaccine incorporating BTV-24, to help mitigate the significant economic losses associated with the disease.

## Data Availability

The original contributions presented in the study are included in the article/**Supplementary Material**. Further inquiries can be directed to the corresponding authors.

## Supplementary material

Supplementary Table 1 Clinical reaction index.

Supplementary Table 2 Gross lesions scoring system.

Supplementary Table 3 Histopathological scoring system.

Supplementary Table 4 Temperature clinical scoring of BTV-24 infected animals.

Supplementary Table 5 Mouth/facial clinical scoring of BTV-24 infected animals.

Supplementary Table 6 Respiratory clinical scoring of BTV-24 infected animals.

Supplementary Table 7 Sensory clinical scoring of BTV-24 infected animals.

Supplementary Figure 1 Standard curve prepared with 10-fold serial dilutions of transcripts and obtained by regression analysis plotting the threshold cycle values (Ct) vs. the logarithm of the actual starting copy number.
