# Supplementary material for: Pathology and pathogenesis of bluetongue virus serotype 24 during experimental infection in native sheep
Source: Front Cell Infect Microbiol. 2026 Mar 10;16:1710415. doi: 10.3389/fcimb.2026.1710415 (PMC13008964; doi:10.3389/fcimb.2026.1710415)

**Supplementary Table 1 Clinical reaction index**

| **S. No.** | **Name of Score** | **Criteria** | **Score** |
| --- | --- | --- | --- |
| 1. | Temperature score | ≤39.0℃ | 0 |
|  |  | ≥39.1℃ | 1 |
|  |  | ≤40.0℃ | 2 |
|  |  | - ≥40.1℃ | 3 |
|  |  | ≥41.1℃ | 4 |
| 2. | Mouth/facial lesion score | Absence of clinical signs | 0 |
|  |  | Congestion of conjunctival and buccal mucosa | 1 |
|  |  | Presence of erosion or ulcers | 2 |
|  |  | Cyanosis of tongue | 3 |
|  |  | Edema of face | 4 |
| 3. | Respiratory score | Severity of nasal discharge: Serous to bloody discharge and respiratory distress | Scale: 0–4 |
| 4. | Sensory score | Anorexia and depression | 1 point/ day |

**Supplementary Table 2 Gross lesions scoring system**

| **S. No.** | **Organ** | **Lesion** | **Score** | |
| --- | --- | --- | --- | --- |
| 1. | Skin and skeletal muscle | Subcutaneous oedema | Facial | 1 pt |
|  |  |  | Nasal | 1 pt |
|  |  |  | Lips | 1 pt |
|  |  |  | Mandibular | 1 pt |
|  |  |  | Neck | 2 pts |
|  |  | Oedema within the facial planes of the abdominal cavity or hindlimbs | 3 pts | |
| 2. | Cardiorespiratory system | Moderate hydrothorax | 2 pts | |
|  |  | Intense hydrothorax | 3 pts | |
|  |  | Pleural effusion | 1 pt | |
|  |  | Pulmonary haemorrhages | 1 pt | |
|  |  | Pulmonary oedema with froth in the trachea | 3 pts | |
|  |  | Moderate hydropericardium | 2 pts | |
|  |  | Intense hydropericardium | 3 pts | |
|  |  | Pericardial effusion | 1 pt | |
|  |  | Haemorrhages/necrosis on the papillary muscles of the left ventricle | 3 pts | |
|  |  | Sub-intimal haemorrhages in the pulmonary artery | 3 pts | |
|  |  | Sub-intimal haemorrhages in the aorta | 3 pts | |
|  |  | Sub-intimal haemorrhages in other great vessels | 3 pts | |
| 3. | Digestive system | Oesophagus | Vascular congestion | 1 pt |
|  |  |  | Haemorrhages | 1 pt |
|  |  |  | Erosions/ulcers | 1 pt |
|  |  | Forestomach | Vascular congestion | 1 pt |
|  |  |  | Haemorrhages | 1 pt |
|  |  |  | Erosions/ulcers | 1 pt |
|  |  | Ileum | Hyperaemia | 1 pt |
|  |  |  | Mucosal oedema | 1 pt |
|  |  |  | Haemorrhages | 1 pt |
|  |  | Ileocaecal valve | Hyperaemia | 1 pt |
|  |  |  | Mucosal oedema | 1 pt |
|  |  |  | Haemorrhages | 1 pt |
|  |  | Liver | Vascular congestion | 1 pt |
|  |  |  | Haemorrhages | 1 pt |
|  |  | Pancreas | Haemorrhages- | 1 pt |
| 4. | Lymphoid system | Spleen | Enlarged | 1 pt |
|  |  |  | Reactive white pulp | 1 pt |
|  |  | Prescapular, submandibular, retropharyngeal, tracheobronchial, ileocaecal, and renal lymph nodes | Enlarged and oedematous | 1 pt |
|  |  |  | Haemorrhages | 1 pt |
| 5. | Urinary system | Kidneys | Hyperaemia | 1 pt |
|  |  |  | Oedema | 2 pts |
|  |  |  | Haemorrhages | 1 pt |
|  |  |  | Other changes | 1 pt |
|  |  | Urinary bladder | Hyperaemia | 1 pt |
|  |  |  | Oedema | 2 pts |
|  |  |  | Haemorrhages | 1 pt |
| 6. | Central nervous system | | Hyperaemic meninges | 1 pt |
|  |  |  | Oedema | 2 pts |
|  |  |  | Haemorrhages | 2 pts |

**Supplementary Table 3 Histopathological scoring system**

| **S. No.** | **Criteria** | **Score** |
| --- | --- | --- |
| 1. | Absence of lesions | 0 |
| 2. | Mild congestion | 1 |
| 3. | Severe congestion, mild hemorrhages, and infiltration of inflammatory cells | 2 |
| 4. | Moderate hemorrhage, oedema, and infiltration of inflammatory cells | 3 |
| 5. | Severe hemorrhage, oedema, and infiltration of inflammatory cells. | 4 |

**Supplementary Table 4 Temperature clinical scoring of BTV-24 infected animals**

| **Animal No.** | **Days post-inoculation (DPI)** | | | | | | | | | | | | | | | | **Total score** |
| --- | --- | --- | --- | --- | --- | --- | --- | --- | --- | --- | --- | --- | --- | --- | --- | --- | --- |
|  | **1** | **2** | **3** | **4** | **5** | **6** | **7** | **8** | **9** | **10** | **11** | **12** | **13** | **14** | **15** | **16** |  |
| BT24-1 | 0 | 0 | 0 | 1 | 1 | 1 | 1 | 1 | 0 | 0 | 0 |  |  |  |  |  | **5** |
| BT24-2 | 0 | 0 | 0 | 1 | 1 | 1 | 1 |  |  |  |  |  |  |  |  |  | **4** |
| BT24-3 | 0 | 1 | 0 | 0 | 2 | 1 | 1 | 1 | 1 | 1 | 1 | 1 | 1 | 1 | 0 | 1 | **12** |
| BT24-4 | 0 | 0 | 0 | 1 |  |  |  |  |  |  |  |  |  |  |  |  | **1** |
| BT24-5 | 0 | 0 | 0 | 0 | 0 | 1 | 0 | 0 | 0 | 0 | 0 | 1 | 0 | 0 | 1 | 0 | **3** |
| BT24-6 | 1 | 1 | 2 | 1 | 1 | 1 | 1 | 1 | 1 | 1 | 1 | 1 | 1 | 1 | 1 | 1 | **17** |
| **Average score** | 0.17 | 0.33 | 0.33 | 0.67 | 1 | 1 | 0.75 | 0.75 | 0.5 | 0.5 | 0.5 | 1 | 0.67 | 0.67 | 0.67 | 0.67 | **10.18** |

Total score: 42; Average cumulative score: 10.18

**Supplementary Table 5 Mouth/facial clinical scoring of BTV-24 infected animals**

| **Animal No.** | **Days post-inoculation (DPI)** | | | | | | | | | | | | | | | | **Total score** |
| --- | --- | --- | --- | --- | --- | --- | --- | --- | --- | --- | --- | --- | --- | --- | --- | --- | --- |
|  | **1** | **2** | **3** | **4** | **5** | **6** | **7** | **8** | **9** | **10** | **11** | **12** | **13** | **14** | **15** | **16** |  |
| BT24-1 | 0 | 1 | 1 | 1 | 1 | 1 | 0 | 0 | 0 | 0 | 0 |  |  |  |  |  | **5** |
| BT24-2 | 0 | 0 | 3 | 2 | 2 | 1 | 2 |  |  |  |  |  |  |  |  |  | **10** |
| BT24-3 | 0 | 1 | 1 | 0 | 1 | 0 | 1 | 0 | 0 | 0 | 1 | 1 | 0 | 0 | 0 | 0 | **5** |
| BT24-4 | 0 | 0 | 0 | 1 |  |  |  |  |  |  |  |  |  |  |  |  | **0** |
| BT24-5 | 0 | 0 | 0 | 1 | 2 | 1 | 0 | 0 | 0 | 0 | 0 | 0 | 0 | 1 | 0 | 0 | **4** |
| BT24-6 | 0 | 0 | 1 | 1 | 0 | 0 | 0 | 0 | 0 | 0 | 0 | 0 | 0 | 0 | 0 | 0 | **2** |
| **Average score** | 0 | 0.33 | 1 | 1 | 1.2 | 0.6 | 0.6 | 0 | 0 | 0 | 0.25 | 0.33 | 0 | 0.33 | 0 | 0 | **5.64** |

Total score: 26; Average cumulative score: 5.64

**Supplementary Table 6 Respiratory clinical scoring of BTV-24 infected animals**

| **Animal No.** | **Days post-inoculation (DPI)** | | | | | | | | | | | | | | | | **Total score** |
| --- | --- | --- | --- | --- | --- | --- | --- | --- | --- | --- | --- | --- | --- | --- | --- | --- | --- |
|  | **1** | **2** | **3** | **4** | **5** | **6** | **7** | **8** | **9** | **10** | **11** | **12** | **13** | **14** | **15** | **16** |  |
| BT24-1 | 1 | 0 | 0 | 1 | 2 | 1 | 1 | 1 | 0 | 0 | 0 |  |  |  |  |  | **7** |
| BT24-2 | 0 | 0 | 0 | 0 | 0 | 0 | 1 |  |  |  |  |  |  |  |  |  | **1** |
| BT24-3 | 1 | 0 | 0 | 0 | 0 | 0 | 0 | 0 | 0 | 0 | 0 | 1 | 0 | 0 | 0 | 0 | **2** |
| BT24-4 | 0 | 0 | 0 | 0 |  |  |  |  |  |  |  |  |  |  |  |  | **0** |
| BT24-5 | 1 | 1 | 1 | 0 | 2 | 2 | 1 | 0 | 0 | 0 | 0 | 0 | 0 | 0 | 0 | 0 | **8** |
| BT24-6 | 0 | 1 | 1 | 1 | 1 | 0 | 0 | 0 | 0 | 1 | 0 | 0 | 0 | 0 | 0 | 0 | **5** |
| **Average score** | 0.5 | 0.33 | 0.33 | 0.33 | 1.25 | 0.6 | 0.6 | 0.25 | 0 | 0.25 | 0 | 0.33 | 0 | 0 | 0 | 0 | **4.77** |

Total score: 23; Average cumulative score: 4.77

**Supplementary Table 7 Sensory clinical scoring of BTV-24 infected animals**

| **Animal No.** | **Days post-inoculation (DPI)** | | | | | | | | | | | | | | | | **Total score** |
| --- | --- | --- | --- | --- | --- | --- | --- | --- | --- | --- | --- | --- | --- | --- | --- | --- | --- |
|  | **1** | **2** | **3** | **4** | **5** | **6** | **7** | **8** | **9** | **10** | **11** | **12** | **13** | **14** | **15** | **16** |  |
| BT24-1 | 0 | 0 | 0 | 1 | 0 | 0 | 0 | 0 | 0 | 0 | 0 |  |  |  |  |  | **1** |
| BT24-2 | 0 | 0 | 0 | 1 | 0 | 0 | 0 |  |  |  |  |  |  |  |  |  | **1** |
| BT24-3 | 0 | 0 | 0 | 0 | 1 | 0 | 1 | 0 | 0 | 0 | 0 | 0 | 0 | 0 | 0 | 0 | **2** |
| BT24-4 | 0 | 0 | 0 | 0 |  |  |  |  |  |  |  |  |  |  |  |  | **0** |
| BT24-5 | 0 | 0 | 0 | 0 | 0 | 0 | 0 | 0 | 0 | 0 | 0 | 0 | 0 | 0 | 0 | 0 | **0** |
| BT24-6 | 0 | 0 | 0 | 0 | 1 | 0 | 0 | 0 | 0 | 1 | 0 | 0 | 0 | 0 | 0 | 0 | **2** |
| **Average score** | 0 | 0 | 0 | 0.33 | 0.4 | 0 | 0.2 | 0 | 0 | 0.25 | 0 | 0 | 0 | 0 | 0 | 0 | **1.18** |

Total score: 6; Average cumulative score: 1.18

**Supplementary Figure 1** Standard curve prepared with 10-fold serial dilutions of transcripts and obtained by regression analysis plotting the threshold cycle values (Ct) vs. the logarithm of the actual starting copy number.


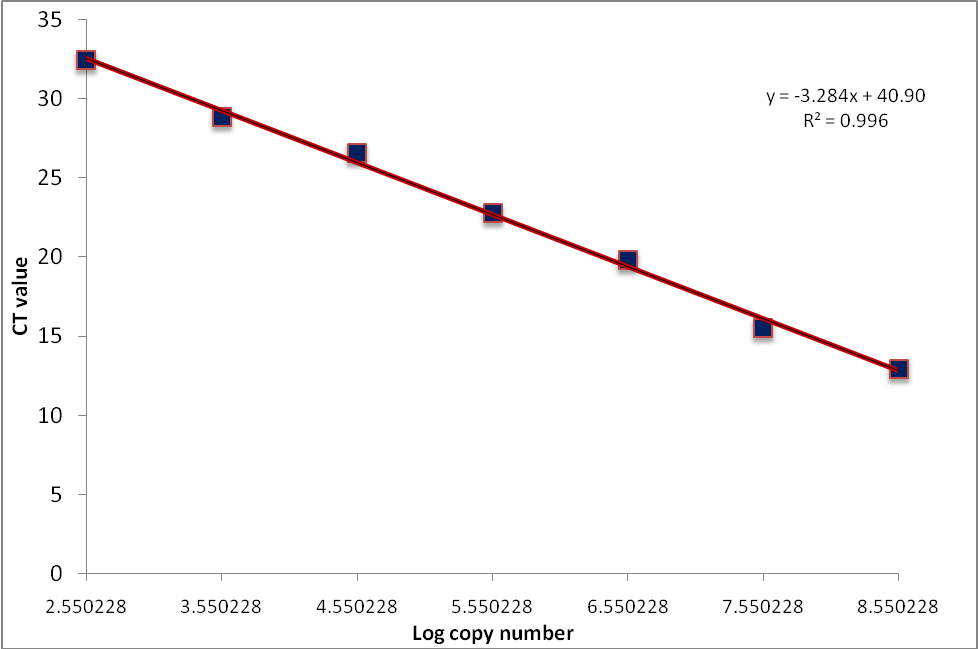

Supplement: Supplementary Table 1 — Clinical reaction index. [file Table1.docx]
